# Generic Sensor Failure Modeling for Cooperative Systems

**DOI:** 10.3390/s18030925

**Published:** 2018-03-20

**Authors:** Georg Jäger, Sebastian Zug, António Casimiro

**Affiliations:** 1 Otto-von-Guericke Universität Magdeburg, Embedded Smart Systems, 39106 Magdeburg, Germany; sebastian.zug@ovgu.de; 2 LASIGE, Faculdade de Ciências, Universidade de Lisboa, 1749-016 Lisboa, Portugal; casim@ciencias.ulisboa.pt

**Keywords:** sensor failures, generic failure modeling, dynamically composed systems, cooperative systems, maintaining safety, cyber-physical-systems

## Abstract

The advent of cooperative systems entails a dynamic composition of their components. As this contrasts current, statically composed systems, new approaches for maintaining their safety are required. In that endeavor, we propose an integration step that evaluates the failure model of shared information in relation to an application’s fault tolerance and thereby promises maintainability of such system’s safety. However, it also poses new requirements on failure models, which are not fulfilled by state-of-the-art approaches. Consequently, this work presents a mathematically defined generic failure model as well as a processing chain for automatically extracting such failure models from empirical data. By examining data of an Sharp GP2D12 distance sensor, we show that the generic failure model not only fulfills the predefined requirements, but also models failure characteristics appropriately when compared to traditional techniques.

## 1. Introduction

Reliable perception of environmental conditions based on (multimodal) sensors is a key feature for autonomously operating applications. However, the mapping process of relevant information from the real world on a digital representation is affected by external and internal disturbances. The different characteristics of possible failures (e.g., continuous or sporadic occurrence, value disturbance with constant, variable or correlated amplitude, absence of value) complicate the development of safety-oriented applications. In this case, engineers have to identify all disturbances that may possibly occur and evaluate their effect on the system’s properties. For this purpose, approaches such as *Failure Mode and Effect Analysis (FMEA)* [1], *Fault Tree Analysis (FTA)* [2], and *Event Tree Analysis (ETA)* [3] are commonly applied. Such methods leverage *failure models* [4] to represent a component’s failure characteristics and thereby support the selection process of appropriate fault tolerance mechanisms at a system’s design-time. At run-time, these tolerance mechanisms limit the effect of component failures and guarantee the system’s compliance with its required safety level. Key to this approach is the assumption that the system’s composition, i.e., its set of components, is determined at design-time and does not change at run-time.

**Figure 1:**
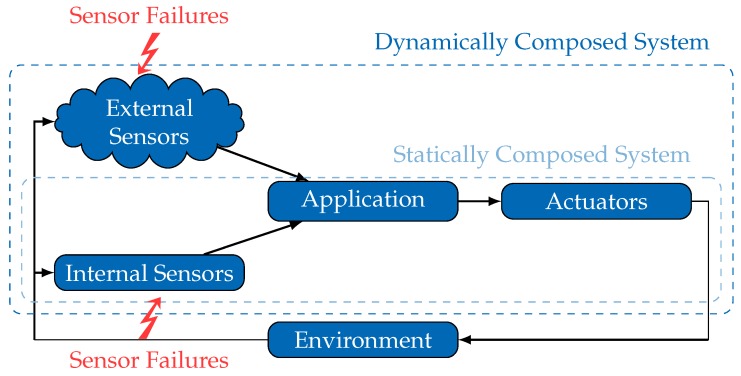
Basic control loop and components of *statically composed systems* and *dynamically composed systems*.

In contrast to such *statically composed systems*, paradigms like Cyber-Physical-Systems [5] and the Internet of Things [6] propose increasing the autonomy of mobile systems by sharing their environmental information and achieving cooperative and/or collaborative behavior. The concept of spatially separated but temporarily integrated external sensors promises an extended coverage and reduces the requirements for local sensors. However, such *dynamically composed systems* (see Figure 1) represent a paradigm shift with respect to safety management and handling. Due to the fact that crucial information, such as failure characteristics of external sensors, are missing during the design phase, a number of engineering tasks (sensor selection, interface adjustment) need to be shifted from design-time to run-time. This specifically affects the safety analysis of external sensors, as the required information of the sensor’s failure characteristics may become available solely at run-time.

**Figure 2:**
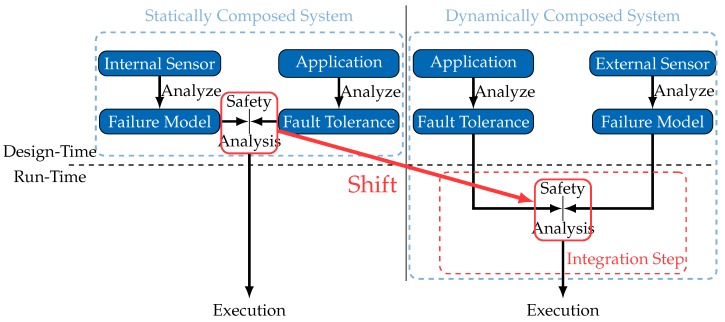
Comparison of safety analysis in *statically composed systems* and *dynamically composed systems*.

Figure 2 illustrates the inevitable shift of part of the safety analysis process to an integration step, which occurs at run-time whenever the system composition is about to change.

A consequence of this shift is that an external sensor is obligated to share not only its observations, but also its failure characteristics. This truly enables dynamically composed systems to conduct a safety analysis at run-time, in a sensor integration step, but requires an explicit and generic model of the sensor failure characteristics. Consequently, the following fundamental questions about such model must be answered. What requirements are posed on failure models of external sensors to be applicable in dynamically composed systems? Are there any suitable failure models already available? If not, how could one construct an appropriate failure model?

In the endeavor of answering these questions, we firstly derive and discuss requirements that have to be fulfilled by failure models to be suitable for such an approach. Then, and considering these requirements, in Section 3, we review the state of the art on sensor failure modeling, which allows for concluding about the lack of appropriate failure models. Therefore, a fundamental contribution of this paper consists in the introduction of a generic failure model fulfilling the initially identified requirements, which we do in Section 4. Furthermore, the second fundamental contribution of this paper is provided in Section 5, where we propose and describe a processing chain to support the automated extraction of a parametrized failure model from raw sensor data. To evaluate this contribution, in Section 6, we conduct experimental evaluations on raw data produced by a Sharp GP2D12 (Sensor manufactured by Sharp Cooperation, Osaka, Japan) [7,8] infrared distance sensor. The evaluation results allow not only to conclude that the processing chain is able to adequately generate generic failure models, but also that the generated failure models indeed fulfill the requirements to be applicable in *dynamically composed systems*. The paper is concluded in Section 7, where the key contributions are summarized and possible future lines of work are expressed.

## 2. Identifying Requirements on Failure Models

In the previous section, we clarified the need for explicit and generic sensor failure models in a dynamically composed system in order to maintain their safety when incorporating external sensors. As this differs from the traditional use of failure models in statically composed systems, different requirements also have to be fulfilled, which we identify in this section.

As shown in Figure 2, a general approach for safety analysis in dynamically composed systems involves an integration step for each new external sensor whose data the application wants to use. A safety analysis is performed in this integration step, using the explicitly made available failure model of the external sensor. Given that the safety analysis performed in the integration step is completely in the hands of the application developer, one should not assume that a specific safety analysis methodology will be used. For generality, the widest possible range of safety analysis mechanisms should be applicable, each potentially implementing different failure representations. A *Generality* requirement is thus expressed as follows.

**Generality:** An appropriate failure model is required to have a generic approach to the representation of failure characteristics. This shall enable an application independent description of failure characteristics that can be transformed into an application specific representation when needed.

The *Generality* requirement ensures that a failure model is defined independently of a specific application. Further to that, for a successful deployment of the proposed scheme (Figure 2) in various types of systems, an appropriate failure model has to be capable of representing failures of a wide range of sensor types (1D, 2D, 3D) to satisfy the needs of both sensor manufacturers and system engineers. Additionally, as external sensors may be virtual sensors [9] or smart sensors [10], they may provide not only raw sensor data, but also high-level features. Consequently, they may be affected by failures in a multitude of different ways. Due to this diversity of sensors and sensor failure types, a *Coverage* requirement is thus defined as follows.

**Coverage:** An appropriate failure model must be capable of representing various failure characteristics in a versatile way.

Both of these requirements (*Generality* and *Coverage*) guarantee the applicability of a failure model to a broad set of systems and scenarios. However, for supporting its intended usage for safety analysis within an integration step, a third requirement ensuring an unambiguous interpretation of a failure model has to be fulfilled. A failure model transferred from an external sensor to an application can only be correctly analyzed when its interpretation is clear. This means that the failure characteristics described by the sensor’s manufacturer need to be extractable from the failure model unambiguously. Otherwise, an application could underestimate the severity of an external sensor’s failure characteristics and compromise safety. A *Clarity* requirement for the representation of a failure model is defined as follows.

**Clarity:** The means used in a failure model to represent failure characteristics must be such that these characteristics will be interpreted unambiguously.

Fulfilling the previous requirements ensures that a semantically correct safety analysis of external sensors is possible. However, being done at run-time in a specific integration step, a final requirement related to its performance must be defined. In fact, this analysis may be subject to temporal restrictions (e.g., in Car-2-Car [11] communication scenarios) or to computing resource restrictions (e.g., in the context of embedded robotic systems). Depending on the application and its context, it should be possible to perform the integration of external sensors in different ways, balancing the cost and the detail of the safety analysis in a suitable manner. In other words, when comparing the failure characteristics of an external sensor with the application needs, it should be possible to do this comparison with different degrees of detail, and naturally also with different degrees of performance and accuracy. A *Comparability* requirement is thus defined as follows.

**Comparability:** For the flexible use of a failure model when comparing failure characteristics and application needs, the representation of failure characteristics must allow for interpretations with various levels of granularity.

In summary, fulfilling the presented requirements ensures the applicability of a failure model to the proposed safety analysis at run-time, in a specific integration step.

## 3. State of the Art on Sensor Failure Modeling

The previous section was dedicated to defining requirements on failure models ensuring their applicability to maintain safety in dynamically composed systems. In this section, we seek to answer the question on whether, and to which extent, existing failure models fulfill these requirements. In this endeavor, we review approaches from the field of Fault Injection, Sensor Networks and Fault Detection and Isolation. Additionally, we consider work from the field of Depth-Cameras to cover yet another sensor type that is not considered in the other three fields. The results of our state-of-the-art review are summarized in Table 1 and explained in detail along the following subsections.

### 3.1. Failure Modeling for Fault Injection

The area of Fault Injection is mainly concerned with simulation-based analysis of a system’s safety [13] or its dependability properties [12]. Due to its simulative approach, failure characteristics of not only sensors, but of system components in general, have to be modeled as realistically as possible to obtain reliable results.

One recently emerged tool in this area is called *ErrorSim* and was introduced by Saraoğlu et al. [12]. The tool is implemented and focused on MATLAB/Simulink (Software produced by Mathworks, Natick, MA, USA) [25]. It enables engineers to mark components of their system model that are either critical or can exhibit faulty behaviors. Individual failure models can be specified for these components and used for fault injection at simulation-time. To support developers during this specification, a number of failure types are predefined: Offset, Stuck-at, and Noise. These are motivated by the IEC 61508 (Standard published by International Electrotechnical Commission (IEC), Geneva, Switzerland) [26], which is a standard for developing safety-critical, electric components.

Although the approach of Saraoğlu et al. [12] allows engineers to define failure models independently of specific applications, their tool *ErrorSim* is closely coupled with the MATLAB/ Simulink framework. Therefore, the *Generality* requirement is only partially fulfilled. The *Coverage* requirement is partially fulfilled too, as the approach covers a wide range of sensor types, due to its goal of analyzing all system components, but restricts the number of representable failure types to three. On the other hand, this enables a clear interpretation of the specified model. As the failure types are defined through mathematical parameters and distributions to facilitate fault injection, the *Clarity* requirement is fulfilled. This also allows the *Comparability* requirement to be fulfilled, since the failure model is represented by a set of mathematical parameters that allow a versatile comparison of failure characteristics with application requirements.

Another approach, proposed by Reiter et al. [13], uses SystemC [27] and C/C++. These authors argue for reusing already developed models of a system for a simulation-based safety analysis. For that, an available SystemC model has to be extended with Fault Injectors and a *Stressor*. The *Stressor* interprets an XML-based description of a failure model and coordinates the Fault Injectors that perform the actual fault injection. In contrast with the work of Saraoğlu et al. [12], the failure model uses the *Behavioral Threat Model (BTM)* [28], a Timed Automata extended with features to manipulate states and variables of the simulated system. In this way, more complex failure characteristics can be represented as engineers are not restricted to predefined failure types, and the *Coverage* requirement is fulfilled. As a downside of this flexible approach, the interpretation of a failure model is not straightforward due to requiring a parsing step, and the *Clarity* requirement is only partially fulfilled. This also causes the *Comparability* requirement not to be fulfilled as the XML-based failure model prevents a direct comparison with other failure models or with application requirements. Finally, and similarly to Saraoğlu et al. [12], this framework is independent of a specific system or application, but is tightly integrated with SystemC. Therefore, the *Generality* requirement is only partially fulfilled.

In summary, since Fault Injection aims at simulations, existing approaches are closely coupled with simulation environments, leading to a partial fulfillment of the *Generality* requirement. Moreover, the failure models are either complex and hence they fulfill the *Coverage* requirement but lack *Clarity*, or are simple, thus satisfying *Clarity* but not *Coverage*.

### 3.2. Failure Modeling in Sensor Networks

Sensor Networks form another field of research requiring appropriate representations of sensor failure characteristics. In general, a sensor network comprises a set of spatially distributed Sensor Nodes, connected either wired or wireless, to observe certain phenomena [29]. Due to external interferences, sensor measurements may be imprecise, leading to inaccurate observations.

Regarding such deviations, Ni et al. [14] distinguish eight categories of sensor failure types commonly seen in sensor networks (e.g., Outlier, Drift, Noise). After providing a general, linguistic description of these failure types, they suggest implementing corresponding detection and filtering algorithms using *features*. These might be signal properties, such as variance and mean, but also analytic metrics, such as a signal’s gradient. Considering datasets from different chemical sensors (ammonia, chlorophyll, CO${}_{2}$, chloride) and sensors observing humidity, temperature and light intensity, Ni et al. [14] exemplarily model the listed sensor failures using the proposed features. While this fulfills the *Coverage* requirement, as a wide range of sensor types and failure types are considered, the approach lacks *Clarity* as no failure model is defined and, instead, individual failure types are modeled. This also causes the *Generality* requirement to be partially fulfilled, since failure types are described only in linguistic terms, while examples for modeling these are application dependent. The *Comparability* requirement is also only partially fulfilled due to the lack of a definite failure model.

Sharma et al. [15] aim at analyzing the prevalence of sensor failures in datasets acquired by real world deployments of sensor networks. They consider three failure types: Short, Noise and Constant. Occurrences of each failure type are detected through four different failure detection algorithms, whose effectiveness is evaluated through fault injection experiments. Due to this approach, mathematical definitions for Noise and Short failures are presented, supporting the fulfillment of the *Clarity* requirement. However, as the Constant failure type is defined solely linguistically, the requirement is only partially fulfilled. This also causes the *Comparability* requirement to be only partially fulfilled, given that only Noise and Short failure types are comparable through their mathematical parameters. The *Generality* requirement is fulfilled because failure types are derived from datasets of four different sensor networks. However, the *Coverage* requirement is only partially fulfilled because only three failure types are defined.

Elnahrawy and Nath [16] are only concerned with the Noise failure type. They propose a filter to clean noisy sensor readings of wireless sensor networks based on the Bayes’ theory. For this, they assume that Noise is always distributed normally with zero mean and a certain variance. As this approach aims at sensor networks in general, its failure model is application independent and thereby fulfills the *Generality* requirement. However, as only Noise failures are represented and they are restricted to a normal distribution, the *Coverage* requirement is not fulfilled. On the other hand, restricting Noise to a Gaussian distribution renders the failure model interpretation fully clear and supports its *Comparability*.

While Elnahrawy and Nath [16] only consider Noise failures, the approach of Sheng et al. [17] is restricted to Outliers. For the detection of Outliers, Sheng et al. [17] provide two distance-based definitions of Outliers. While these are general, supporting the *Generality* requirement, defining the same failure type contradicts the *Clarity* requirement twice. Furthermore, as only Outliers are considered, the *Coverage* requirement is not fulfilled. Finally, and despite the provided definitions being mathematical, the definition of an Outlier depends on the used dataset and hence *Comparability* is not fulfilled.

In contrast to both previous works, the approach of Urteaga et al. [18] aims at providing a distributed scheme for detecting multiple failure types in data of wireless sensor networks, like Noisy Readings, Readings Not in the sensor’s Linear Detection Range (NLDR) (i.e., outside its calibration range), Out of Range Readings (beyond the total detection range of the sensor) or Stuck Readings. To detect these failures, the mean and variance of a series of sensor readings are calculated and checked with adjustable thresholds. The *Generality* and *Coverage* requirements are only partially fulfilled because the description of sensor failures is strongly determined by the considered failure detection scheme and because they are modeled in relation to only mean and variance values, which is limitative. Furthermore, the failure types are defined only linguistically or implicitly through the parameterization of the failure detection scheme. Both do not enable a clear interpretation, leaving the *Clarity* requirement unfulfilled. Finally, due to the unclear definition of the failure types, *Comparability* is not provided either.

In summary, since failure models considered in the field of Sensor Networks are commonly defined in relation to specific datasets, they tend to support the *Generality* requirement but not the *Coverage* one. In addition, whenever linguistic definitions are used, both *Clarity* and *Comparability* are not fulfilled.

### 3.3. Failure Modeling for Fault Detection and Isolation

The field of failure modeling for Fault Detection and Isolation (FDI) targets automated and robotic systems. In this context, failure modeling facilitates the design of processing chains encompassing specialized failure detectors and filters [3] to mitigate sensor failures and countervail their negative effects.

A prominent approach for detecting and filtering sensor failures is generating so-called residual-signals as the difference between observed and predicted sensor values [3]. When no failure is present, this signal is (close to) zero. Deviations from zero indicate sensor failures. Dai et al. [19] use this methodology to define an FDI system for a Pick-and-Place robot, while also defining the failure types Bias, Drift and Complete Failure. For Bias and Drift, Dai et al. [19] provide mathematical equations representing the failure types by specific parameters. However, since they consider Noise as a separate and superimposing failure type, described through a Gaussian distribution, the *Generality* of their approach is reduced. In fact, this is also why the approach proposed by Balaban et al. [20] is considered to only partially fulfill *Generality*. Concerning the *Coverage* requirement, while Dai et al. [19] use specific equations for each failure type, thus limiting representable failure characteristics, Balaban et al. [20] consider a wide range of sensor types (e.g., thermocouples, resistance temperature detectors, piezoelectric sensors) and thus fully satisfy the *Coverage* requirement. In contrast to this, the mathematical definition of failure types in [19] supports the *Clarity* as well as the *Comparability* of the failure model, while the linguistic definitions used in [20] prevent these requirements to be fulfilled.

Heredia et al. [21] present an FDI system for an autonomous helicopter, defining a sensor failure model covering five failure types: Total sensor failure, Stuck with constant bias sensor failure, Drift, Multiplicative-type sensor failure and Outlier. These failure types were defined based on an examination of observations of different types of sensors, namely gyroscopes, accelerometers, magnetic sensors and GPS sensors. Therefore, the *Coverage* requirement is fulfilled. In addition, since the description of the failure model does not depend on the application to an autonomous helicopter or to an FDI system, the *Generality* requirement is also fulfilled. However, the failure types are described only linguistically leaving the *Clarity* requirement unfulfilled. Similarly, as the approach lacks a clear definition of the failure model, *Comparability* is not provided.

**Figure 3:**
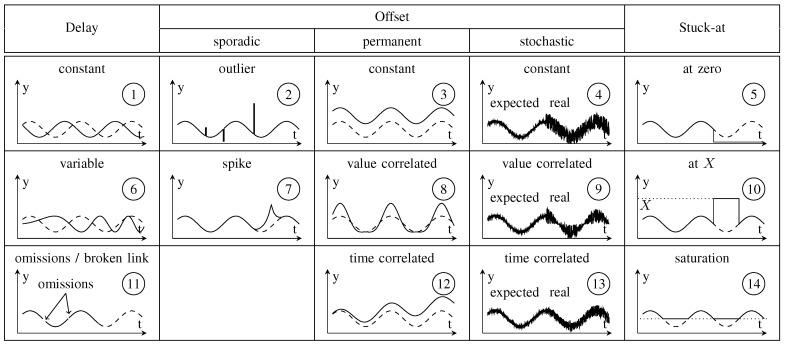
Sensor failure model defined by Zug et al. [4].

Zug et al. [4] provide a detailed failure model in which 14 different failure types are defined (see Figure 3) and categorized into Delay, Offset and Stuck-at failures. In contrast to previously presented failure models, Zug et al. [4] define failure types that may exhibit time- and/or value-correlated failures. The failure amplitude of such failure types might vary depending on the actual sensor value or the operation time. Due to this detailed definition of failure types, the *Coverage* requirement is fulfilled. Likewise, the failure model is defined independently of a certain application, providing general failure types and thereby fulfilling the *Generality* requirement. In contrast to this, the definition itself is linguistic, leaving the *Clarity* and the *Comparability* requirements unfulfilled.

By summarizing the approaches from the field of Fault Detection and Isolation, it becomes apparent that a wide range of sensors and sensor failures are respected when defining failure models (the *Coverage* requirement is fulfilled in most cases, see Table 1), but their definition is either implicit or linguistic, which contradicts the *Clarity* requirement and prevents the *Comparability* requirement from being fulfilled too.

### 3.4. Failure Modeling for Depth Cameras

Although a wide range of sensor types is covered by the approaches that were mentioned so far, none of them considered depth cameras. They are used in state-of-the-art robotic and autonomous systems as they enable observing a broad area of a system’s vicinity [30]. Depending on their working principle, depth cameras are categorized as Time-of-Flight (ToF) cameras and triangulation-based cameras [24]. Regardless of their working principle, their measurements are impaired by sensor failures too.

Foix et al. [22] survey ToF cameras and map sources of errors to the common failure types Noise, Offset, and, specific to depth cameras, Illumination Artifacts [24]. In contrast to (most of) the aforementioned approaches, the authors describe the amplitudes of failures of depth cameras to be distance related. For instance, Offset failures caused by depth distortion or Noise failures caused by non-uniform illumination of the scene depend on the actual distance. To represent these, Foix et al. [22] propose multiple modeling strategies. For Offset failures, B-splines are proposed as the actual failure magnitude follows a sinusoidal curve. Furthermore, Look-up Tables as well as polynomials with three to six degrees are proposed. Finally, Illumination Artifacts are described to be caused, for instance, by multiple light reception. Although the work of Foix et al. [22] aims at ToF cameras, which limits the fulfillment of the *Generality* requirement, failure types common to other sensors are listed. Therefore, *Generality* is partially fulfilled. Similarly, the *Coverage* requirement is partially fulfilled because the overall set of considered failure types is limited. Furthermore, the failure types are solely linguistically defined, leaving both the *Clarity* and the *Comparability* requirements unfulfilled.

In contrast to the work of Foix et al. [22], Khoshelham and Elberink [23] aim at triangulation-based depth cameras, specifically Microsoft’s Kinect camera. They found that the camera exhibits Outlier and Gap (invalid or missing depth measurements) failures, which can be caused by problematic lighting conditions. In the case of Outlier failures, occlusions or shadows are possible sources too. As done in [22], Khoshelham and Elberink [23] describe a distance related Noise in the depth measurements. However, as a Gaussian distribution is assumed for the Noise failure, its contribution to the *Coverage* requirement is limited. Furthermore, as the set of considered failure types is limited, this requirement is only partially fulfilled. The *Generality* requirement is also partially fulfilled because a single triangulation-based RGB-D camera is targeted. As the model itself is described linguistically, with the exception of Noise, the approach lacks *Clarity* and *Comparability*.

Considering not only a specific type of depth cameras, but the most commonly used types (triangulation- based using structured light, triangulation-based using stereo vision, time-of-flight), Höbel et al. [24] aim not only at defining a general failure model for depth cameras, but also at using it to apply the Validity Concept [31] to this sensor type. With this motivation, works on error sources of depth cameras are reviewed in order to define a failure model comprising Noise, Outlier, Offset, Illumination Artifact and Gap failure types. On the one hand, defining the failure model with respect to a wider range of sensor types allows the *Coverage* requirement to be partially fulfilled. On the other hand, as the failure model is defined for application within the Validity Concept, it lacks *Generality*. Furthermore, as the Gap and Offset failure types are solely linguistically defined, *Clarity* as well as *Comparability* are not provided.

In summary, although the field of Depth Cameras address a specific type of sensors, defined failure types, e.g., Noise and Offset, are common to other fields. However, most approaches of this field take only a limited number of failure types into account. This renders the *Coverage* requirement to be only partially fulfilled by all reviewed approaches. Furthermore, failure models are defined linguistically, causing the *Clarity* as well as the *Comparability* requirement to be unfulfilled.

### 3.5. Outstanding Conclusions from the State-of-the-Art Review

From the presented analysis of the state of the art in sensor failure modeling, the following outstanding conclusions can be drawn:

1. No failure model fulfilling all previously identified requirements could be found. The definition of a failure model that fulfills all the requirements, which is done in this paper, is hence a novel and relevant contribution for safety analysis in dynamically composed systems.
2. The *Clarity* requirement is only fully satisfied by a mathematically defined failure model. In fact, as it stands out from Table 1, this requirement is fulfilled only in three cases [12,16,19], whose common denominator is a mathematically defined failure model. Moreover, when the *Clarity* requirement is fulfilled, the *Comparability* requirement is also fulfilled. This is because *Clarity* enables the correct interpretation of a failure model, which facilitates the flexible use of a failure model when comparing failure characteristics with application needs. As a corollary of this conclusion, it is possible to say that, for the purpose of defining a suitable failure model for safety analysis in dynamically composed systems, this failure model must be mathematically defined.
3. *Complexity* of a failure model enables *Coverage* but may jeopardize *Clarity* and *Comparability*. This is observed in five cases [4,14,20,21,28], in which *Coverage* is achieved due to considering a complex approach, but *Clarity* and *Comparability* are not fulfilled. Therefore, the complexity of a failure model has to be balanced between *Coverage* and the requirements of *Clarity* and *Comparability*.

## 4. Introducing a Generic Sensor Failure Model

As observed in the previous section, no failure model fulfilling all predefined requirements could be found in literature. Therefore, in this section, we address the need for such a failure model. We introduce a generic failure model following the conclusions drawn from the state-of-the-art review and aiming at fulfilling the previously defined requirements.

**Figure 4:**
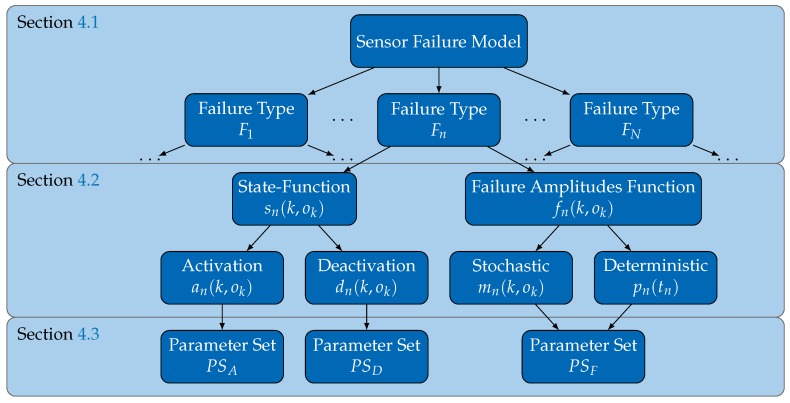
Hierarchical overview on the generic failure model.

Figure 4 illustrates the structure of the failure model, indicating the subsections that address the corresponding components. We start by deriving the general structure of the failure model, using the concept of failure types to decompose a sensor’s failure characteristics (Section 4.1). A consequence is that a failure model is nothing but a set of failure types, each represented through a set of mathematical functions. These are introduced and discussed in detail in Section 4.2. Section 4.3 presents our approach for representing the defined functions and Section 4.4 highlights the reasons why the proposed generic failure model fulfills the requirements identified in Section 2.

### 4.1. Modeling Failure Amplitudes by Failure Types—A Decomposition

Sensors observe a continuous phenomenon e(t)∈R with a sampling period of $T_{s}$ to produce a discrete time series $o_{k}=e\left(k\cdot T_{s}\right)$, where $k\in N_{0}$ is the discrete time index [19,20,32,33]. Accordingly, a sensor maps the magnitude of e(t) to a digital representation $o_{k}$. However, disturbances interfere with the mapping process and cause the sensor to produce impaired sensor observations ${\hat{o}}_{k}$ instead of the theoretically correct observations $o_{k}$. The differences between these values form a series of sensor failure amplitudes $f(k,o_{k})$:

(1) $$f\left(k,o_{k}\right)={\hat{o}}_{k}-o_{k}.$$

In general, a series of failure amplitudes represents a sensor’s behavior in case of a failure and thereby is an instantiation of its failure characteristics. As this behavior can be time- or value-correlated [4,19,32], the function value of $f(k,o_{k})$ depends on the sensor’s operation time *k* as well as on the magnitude of the observed phenomenon $o_{k}$.

The purpose of a failure model is to represent the failure characteristics of a sensor. A common approach to that is to utilize the concept of failure types [3,4,33,34,35]. By applying this concept, failure characteristics are decomposed into a set of *N* independent failure types, each representing a distinct property of the overall failure characteristics. As the failure amplitudes $f(k,o_{k})$ are instantiations of such failure characteristics, they can be decomposed into *N* failure types too:

(2) $$f\left(k,o_{k}\right)=\sum_{n=1}^{N}\underset{n-\mathrm{th}\;\mathrm{Failure}\;\mathrm{Type}\;(F_{n}).}{\underset{︸}{s_{n}\left(k,o_{k}\right)\cdot f_{n}\left(k,o_{k}\right)}}$$

For this decomposition, we assume that a failure type consists of two basic elements: $f_{n}\left(k,o_{k}\right)$ and $s_{n}\left(k,o_{k}\right)$.

$f_{n}\left(k,o_{k}\right)\;\in \;R$: This function models the aspect of the overall failure characteristics that the *n*-th failure type represents. In other words, as we decompose the initial failure amplitudes
$f(k,o_{k})$ into *N* failure types, each failure type has to contribute a series of failure amplitudes
$f_{n}\left(k,o_{k}\right)$ of its own. This series, however, contains only the aspect of the overall failure characteristics that the *n*-th failure type represents.

$s_{n}\left(k,o_{k}\right)\in \left\{0,1\right\}$: Given that a failure type does not always contribute to the failure amplitude, this state-function models the activity and inactivity of the failure type. Its value-range is limited to the set {0,1}, where 0 indicates inactivity and 1 indicates activity of the *n*-th failure type.

In summary, Equation (2) states the basic structure of our generic failure model. A sensor’s failure characteristics, represented by its failure amplitudes $f(k,\;o_{k})$, is modeled by a set of failure types $\{F_{1},\;$…$,\;F_{N}\}$, where *N* is the total number of different characteristics. Each failure type is represented by its state-function $s_{n}\left(k,\;o_{k}\right)\in \left\{0,\;1\right\}$, describing whether the *n*-th failure type is active ($s_{n}\left(k,\;o_{k}\right)=1$) or inactive ($s_{n}\left(k,\;o_{k}\right)=0$) at time *k*, and its failure amplitudes $f_{n}\left(k,\;o_{k}\right)\in R$, describing a distinct aspect of a sensor’s failure characteristics. Thus, while the state-function $s_{n}\left(k,\;o_{k}\right)$ models when the failure type contributes to the overall failure amplitude, its failure amplitudes $f_{n}\left(k,\;o_{k}\right)$ models how the failure type contributes to the overall failure amplitude.

### 4.2. Elements of a Failure Type

This section provides concrete solutions for constructing the two functions that represent each failure type, $f_{n}\left(k,\;o_{k}\right)$ and $s_{n}\left(k,\;o_{k}\right)$. We firstly introduce a scheme for representing time- and value-correlated random distributions, as this is required for both functions. Then, we focus on a failure type’s failure amplitudes (Section 4.2.2) and, finally, we address a failure type’s state function (Section 4.2.3).

#### 4.2.1. Representing a Time- and Value-Correlated Random Distribution

Due to the stochastic nature of failure amplitudes and their possible time- and value-correlated magnitudes [4,19,32], a general methodology for representing time- and value-correlated random distributions is required.

In this endeavor, we presume that the time- and value-correlations of a random variable *Y* affect only the mean $\mu_{Y}$ and standard deviation $\sigma_{Y}$ of its underlying random distribution. Therefore, $\mu_{Y}\left(k,o_{k}\right)\in R$ and $\sigma_{Y}\left(k,o_{k}\right)\in R$ are functions of time *k* and value $o_{k}$ while the uncorrelated random distribution is given by $D_{Y}^{-1}\left(x\right),\;x\in U\left(0,1\right)$, which is the inverse cumulative distribution function (ICDF), also called quantile function [36]. By applying the inverse of the well-known Z-score normalization [37], these three elements resemble a time- and value-correlated random distribution as:

(3) $$y\left(k,\;o_{k}\right)=\sigma_{Y}\left(k,\;o_{k}\right)\cdot D_{Y}^{-1}\left(x\right)+\mu_{Y}\left(k,\;o_{k}\right).$$

The ICDF maps a probability *x* to a distribution value $D_{Y}^{-1}\left(x\right)$ for arbitrary distributions [36]. Consequently, it enables sampling the represented distribution by providing uniformly distributed random numbers x∈U(0,1). This property is utilized in Equation (3) to firstly sample a random value from $D_{Y}^{-1}\left(x\right)$ and then multiply it by the time- and value-correlated standard deviation $\sigma_{Y}\left(k,\;o_{k}\right)$. By shifting the value using the also time- and value-correlated mean $\mu_{Y}\left(k,\;o_{k}\right)$, the resulting random value $y(k,\;o_{k})$ follows a time- and value-correlated random distribution. In other words, while the underlying random distribution is modeled by $D_{Y}^{-1}\left(x\right)$ using an ICDF, the time- and value-correlations are captured by $\mu_{Y}\left(k,\;o_{k}\right)$ and $\sigma_{Y}\left(k,\;o_{k}\right)$. It should be noted that using the inverse cumulative distribution function to represent the normalized distribution turns the model into a *generative model* [38].

#### 4.2.2. A Failure Type’s Failure Amplitudes Function

With a concept for representing time- and value-correlated random distributions in place, we now discuss the failure amplitudes function $f_{n}\left(k,\;o_{k}\right)$ in detail.

The purpose of $f_{n}\left(k,\;o_{k}\right)$ is to represent the contribution that a failure type has on a sensor’s failure characteristics. As mentioned before, a failure type is intended to represent only a specific, deterministic aspect of the overall characteristics. On the other hand, failure characteristics are subject to randomness. To account for both properties, $f_{n}\left(k,\;o_{k}\right)$ encompasses two parts: a deterministic, called the failure pattern $p_{n}\left(t_{n}\right)$ [3,33,35], and a stochastic part $m_{n}\left(k,\;o_{k}\right)$. While the deterministic part models the specific aspect of the failure characteristics, the stochastic part represents the randomness with which the aspect may occur. Considering both parts, the function $f_{n}\left(k,\;o_{k}\right)$ is defined as follows:

(4) $$f_{n}\left(k,\;o_{k}\right)=\underset{\mathrm{stochastic}}{\underset{︸}{m_{n}\left(k,\;o_{k}\right)}}\;\;\cdot \underset{\mathrm{deterministic}}{\underset{︸}{p_{n}\left(t_{n}\right)}}.$$

**Figure 5:**
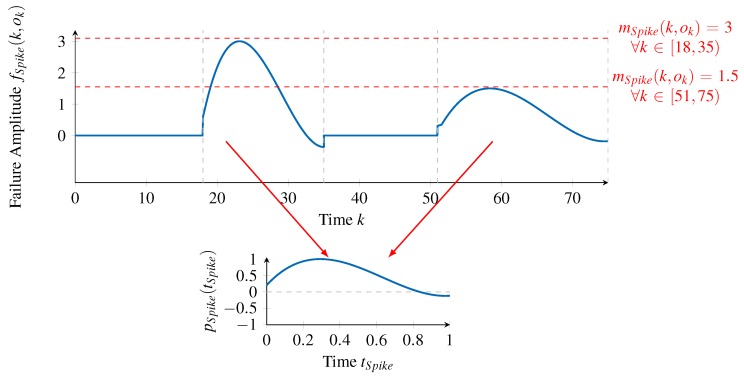
Example of a Spike failure type with two occurrences, modeled through a failure pattern and a stochastic function.

To illustrate how a failure type is composed of a failure pattern $p_{n}\left(t_{n}\right)$ and a stochastic part $m_{n}\left(k,\;o_{k}\right)$, we present in Figure 5 a concrete example of how the failure amplitudes function of a single failure type can look. In this example, we ignore the state-function that models the activation and deactivation of the failure pattern.

For the sake of the example, we name the failure type $F_{Spike}$. A generic failure pattern $p_{n}\left(t_{n}\right)\;\in \;\left[-1,\;1\right]$ with $t_{n}\in \left[0,\;1\right]$ models a normalized shape of the failure amplitude. Consequently, and considering the example in Figure 5, only the failure amplitudes $f_{Spike}\left(k,\;o_{k}\right)\neq 0$ are considered to form $p_{Spike}\left(t_{Spike}\right)$ depicted in the bottom graph. To address the *Coverage* requirement, a failure pattern is normalized in two ways. Firstly, the magnitude of the failure pattern is restricted to the range of [−1,1]. Therefore, $p_{n}\left(t_{n}\right)$ only describes the shape of the failure pattern, but not its actual instantiations. Secondly, the length in the time domain of the pattern is expressed within the range [0,1]. This decouples the failure pattern’s time index $t_{n}$ from the overall time index *k* and thereby enables modeling the length of different instantiations of this failure type separately, namely by the state-function $s_{n}\left(k,\;o_{k}\right)$ (see the next section). While $p_{Spike}\left(t_{Spike}\right)$ represents a spike-like failure pattern in the example of Figure 5, arbitrary curves are possible in general. In the literature, a failure pattern enables a categorization of failure types like, for instance, Outlier, Spike, Offset or Drift [4,14].

Due to its normalized representation, a failure pattern $p_{n}\left(t_{n}\right)$ does not directly form the failure type’s failure amplitudes $f_{n}\left(k,\;o_{k}\right)$ but is scaled by the stochastic part $m_{n}\left(k,\;o_{k}\right)$ (see Equation (4)). Since the scaling might be time- or value-correlated, we apply the previously introduced concept of time- and value-correlated random distributions to represent this part. Therefore, using Equation (3) to represent $m_{n}\left(k,\;o_{k}\right)$ and replacing it in Equation (4) yields the final definition of a failure type’s failure amplitudes function:

(5) $$f_{n}\left(k,\;o_{k}\right)=\underset{\mathrm{stochastic}}{\underset{︸}{\left[\sigma_{n}\left(k,\;o_{k}\right)\cdot D_{n}^{-1}\left(x\right)+\mu_{n}\left(k,\;o_{k}\right)\right]}}\;\;\cdot \underset{\mathrm{deterministic}}{\underset{︸}{p_{n}\left(t_{n}\right)}}.$$

Considering again the example in Figure 5, the stochastic part $m_{Spike}\left(k,o_{k}\right)$ must allow the representation of the two instances in the failure type. This is facilitated by the concept of Section 4.2.1 in different ways: on the one hand, the ICDF facilitates representing a random distribution specific to the Spike failure type ($D_{Spike}^{-1}\left(x\right)$), which accounts for varying scaling values in general. Therefore, the scaling value of the first occurrence is $m\left(k,o_{k}\right)=3.0,\forall k\in \left[18,35\right)$ while the second is $m\left(k,\;o_{k}\right)=1.5,\forall k\in \left[51,75\right)$. On the other hand, this variation could also be represented explicitly by specifying time- and value-correlated functions for the mean $\mu_{Spike}\left(k,\;o_{k}\right)$ and standard deviation $\sigma_{Spike}\left(k,\;o_{k}\right)$.

#### 4.2.3. A Failure Type’s State-Function

While Equation (5) describes the failure amplitudes of a failure type when $f_{n}\left(k,o_{k}\right)\neq 0$, it is necessary to model when the failure type is active ($s_{n}\left(k,o_{k}\right)=1$) or inactive ($s_{n}\left(k,o_{k}\right)=0$). For each new activation of a failure type, we say that there is a new occurrence or instance of the failure type. Commonly, the occurrence of a failure type is modeled by a static occurrence probability [12]. However, such a probability accounts only for the activation of a failure type, that is, when a failure type’s state-function switches from 0 to 1 ($s_{n}\left(k,o_{k}\right)=0,\;s_{n}\left(k+1,o_{k+1}\right)=1$). The deactivation of a failure type is defined either statically, which contradicts the *Coverage* requirement, or implicitly, which contradicts the *Clarity* requirement. Therefore, to fulfill both requirements, we directly model the activation and deactivation of a failure type.

For that, we utilize the concept of (Mean) Time Between Failures (TBF) and (Mean) Time to Repair (TtR) [39]. Traditionally, these are parameters specifying the reliability of a system. The Mean Time Between Failures states the average time between two successive breakdowns of the system while the Mean Time to Repair states the average time it will take to repair a system after a breakdown. However, by deeming a sensor as a system, these concepts can be applied too. In this manner, the Time Between Failures denotes the time between two successive occurrences of a failure type and thereby is a measure of when it becomes active. Complementary to this, the Time to Repair translates to the length of a single occurrence of a failure type and thereby is a measure of when it becomes deactivated.

**Figure 6:**
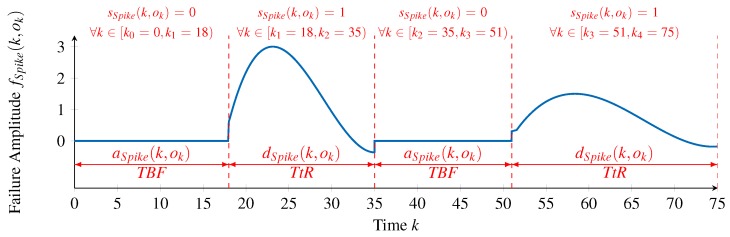
State function for modeling an exemplary Spike failure type with two occurrences.

To illustrate these concepts, we consider once again the example of the Spike failure type, extending it in Figure 6 to show the time intervals during which the failure type is active and inactive. In the example, the first occurrence of the failure type lasts for 17 time units, corresponding to $s_{Spike}\left(k,\;o_{k}\right)\;=\;1,\forall k\in \left[18,35\right)$, while the second occurrence lasts for 24 time units (k∈[51,75)). These time intervals represent two instances of the TtR. The time intervals during which the failure type is not active constitute instances of the TBF, in this case with lengths of 18 time units (k∈[0,18)) and 16 time units (k∈[35,51)).

In summary, the time between two occurrences as well as the length of different occurrences of a failure type may vary stochastically. Therefore, we utilize time- and value-correlated random distributions (see Section 4.2.1) to represent these. In this manner, $a_{n}\left(k,o_{k}\right)$ is defined to represent the TBF, that is, the activation of the *n*-th failure type. Likewise, $d_{n}\left(k,o_{k}\right)$ represents its TtR, that is, the deactivation of the *n*-th failure type. Using Equation (3), they are represented as:

(6) $$\begin{array}{cc} a_{n}\left(k,o_{k}\right) & =\sigma_{a}\left(k,o_{k}\right)\cdot D_{a}^{-1}\left(x\right)+\mu_{a}\left(k,o_{k}\right), \end{array}$$

(7) $$\begin{array}{cc} d_{n}\left(k,o_{k}\right) & =\sigma_{d}\left(k,o_{k}\right)\cdot D_{d}^{-1}\left(x\right)+\mu_{d}\left(k,o_{k}\right). \end{array}$$

To define the state function $s_{n}\left(k,o_{k}\right)$ using Equations (6) and (7), we leverage the fact that both $a_{n}\left(k,o_{k}\right)$ and $d_{n}\left(k,o_{k}\right)$ define intervals that are disjoint to each other:

(8) $$s_{n}\left(k,o_{k}\right)=\left\{\begin{array}{cc} 0, & k\in \left[k_{2i},k_{2i+1}\right),\;\forall i\in N_{0},\\ 1, & k\in \left[k_{2i+1},k_{2i+2}\right),\;\forall i\in N_{0}, \end{array}\right.$$

with the starting condition of $k_{0}=0$ and

(9) $$k_{j}=\left\{\begin{array}{c} k_{j-1}+a_{n}\left(k_{j-1},o_{k_{j-1}}\right),\;j=1,3,5,\ldots ,\\ k_{j-1}+d_{n}\left(k_{j-1},o_{k_{j-1}}\right),\;j=2,4,6,\ldots \end{array}\right.$$

The subscripted time steps $k_{j}$ recursively define the border of the intervals in which the *n*-th failure type is active or inactive. Regarding the exemplary failure type $F_{Spike}$, Figure 6 depicts the output of the state function $s_{Spike}\left(k,o_{k}\right)$ together with the intervals correspondingly defined by the activation function $a_{Spike}\left(k,o_{k}\right)$ and deactivation function $d_{Spike}\left(k,o_{k}\right)$.

### 4.3. Representing Failure Types by Radial Basis Function Networks

The mathematical functions introduced in the previous sections allow describing a failure type in detail. However, representing its functions in terms of actual parameters requires the application of an appropriate function approximation scheme. To address this problem while trying to address the *Coverage*, *Comparability* and *Clarity* requirements, we propose using artificial neural networks, specifically radial basis function networks (RBF networks) [40]. In essence, RBF networks are mathematical functions that provide a deterministic output value for a given input value (e.g., y=rbf(x), where *x* and *y* can be vectors). Internally, matrices of parameters (associated to so-called neurons) determine the represented functions. Given an appropriate number of neurons, RBF networks are proven to be capable of approximating arbitrary functions with arbitrary precision on a closed interval [41], which satisfies the *Coverage* requirement. The parameters encoding a desired function within an RBF network are determined during a process called *training*. Moreover, the proof implies that the level of granularity with which not only a function, but a failure type in general, is represented can be adapted by adapting the number of neurons. In this manner, the *Comparability* is addressed as well. Finally, the matrices of RBF networks can be extracted to reduce the representation of each failure type to three parameter sets: $PS_{A}$ holding the parameters relevant for a failure type’s activation function, $PS_{D}$ holding the parameters relevant for a failure type’s deactivation function, and $PS_{F}$ holding the parameters relevant for a failure type’s failure amplitudes function. As each of these parameters is associated with an RBF network, representing a defined function with a defined interpretation, *Clarity* is supported too.

### 4.4. Generic Failure Model Properties

During the definition of the generic failure model, we considered the requirements identified in Section 2. To check whether these are fulfilled, we review each of them with respect to the generic failure model:

**Generality:** Asking for an application independent failure model, the *Generality* requirement is fulfilled as we did not consider a specific application rather then dynamically composed systems in general while defining the failure model.

**Clarity:** To fulfill this requirement, the generic failure model represents each failure type through a set of defined, mathematical functions, each fully described by specific parameters. As these functions are interpretable in only one way, misinterpretation and ambiguity is prevented.

**Coverage:** This requirement is fulfilled for two reasons: first, the failure model does not limit the number of possible failure types. Thus, even complex failure characteristics can be decomposed into several failure types. Second, the functions for representing a failure type are approximated by RBF networks that are capable of universally approximating functions, even in higher dimensions [41]. As a consequence, all failure characteristics are supported.

**Comparability:** An observation from the state-of-the-art review in Section 3 is the close relation between *Clarity* and *Comparability*. Due to the fulfillment of the former requirement, the generic failure model supports the *Comparability* requirement in general. Furthermore, versatile evaluation strategies of the failure model are possible. The minimal and maximal failure amplitude represented by the model are extractable efficiently, while detailed analyses are supported as well. Finally, the failure model can be efficiently evaluated, in general, through the use of RBF networks. Therefore, this requirement is also fulfilled.

We note that the ability to fulfill all the previously identified requirements is also due to an appropriate balance between the needs of individual requirements. As concluded from the state-of-the-art review in Section 3, the complexity of a failure model may jeopardize one requirement in favor of another. Within the generic failure model, complexity is increased by utilizing the concept of failure types and enabling the representation of their time- and value-correlated occurrences to address *Coverage*. In contrast, each of the defined functions for representing a failure type has a dedicated meaning, improving *Clarity* as well as *Comparability*. Therefore, the complexity of the failure model is balanced between the needs of each requirement.

## 5. Automatic Designation of a Failure Model from Raw Sensor Data

**Figure 7:**
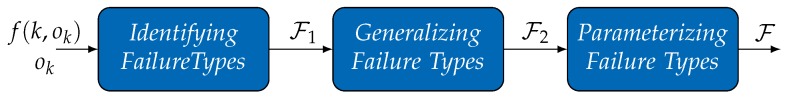
Processing chain for automated failure model generation.

Modeling failure characteristics of real sensors requires finding appropriate failure types by examining empirical observations of the sensor in question. Therefore, in this section, we propose a processing chain for converting a series of failure amplitudes into a parameterized failure model, doing this in an automated manner. An overview on the phases of the processing chain is given in Figure 7.

The processing chain receives as input a series of failure amplitudes $f(k,o_{k})$, calculated by applying Equation (1) to sensor observations and the corresponding reference values $o_{k}$ (ground truth). The reference values $o_{k}$ are also used in the processing chain. These inputs are converted by the processing chain into a set of failure types $F=\{F_{1},\;F_{2},\;\cdots ,\;F_{N}\}$ constituting the generated failure model. The processing chain involves three phases. *Identifying Failure Types* is the first phase, which identifies an intermediate set $F_{1}$ of failure types representing the failure amplitudes $f(k,o_{k})$. We discuss this phase in Section 5.1. Then, Section 5.2 details the second phase, *Generalizing Failure Types*, in which pairs of failure types in $F_{1}$ that are similar to each other are identified and combined into a single failure type. The output of this phase is the set $F_{2}$, which holds all relevant failure types. Finally, Section 5.3 discusses the last phase, *Parameterizing Failure Types*, which uses the gathered information about each failure type in $F_{2}$ to train RBF networks representing each failure types’ functions, as defined in Section 4.2. In this manner, the final set of failure types F is determined.

### 5.1. Identifying Failure Patterns

The first phase of the processing chain exploits Equations (2) and (4), to decompose the failure amplitudes $f(k,o_{k})$ received as input into a set of $N_{1}=\left|F_{1}\right|$ failure types, as follows:

(10) $$f\left(k,o_{k}\right)=\sum_{n=1}^{N_{1}}s_{n}\left(k,o_{k}\right)\cdot m_{n}\left(k,o_{k}\right)\cdot p_{n}\left(t_{n}\right).$$

For each failure type, it is necessary to identify information about its occurrences (time steps at which $s_{n}\left(k,o_{k}\right)=1$), the magnitude of each occurrence (value of $m_{n}\left(k,o_{k}\right)$) and the failure type’s failure pattern ($p_{n}\left(t_{n}\right)$). This information will be passed to the next phase, along with the initial inputs of the processing chain.

In order to obtain this information from the given failure amplitudes, we firstly make two assumptions on the failure types to identify. The first assumption is that all occurrences of a single failure type have the same duration, that is, the same Time to Repair. Although it is known that the Time to Repair of a failure type may vary following a time- and value-correlated random distribution, this assumption simplifies the process of identifying appropriate failure patterns. If two occurrences of a failure type exist with different lengths of duration, in this phase, they will be classified as occurrences of different failure types. The second assumption is that failure patterns are defined only in the range of $p_{n}\left(t_{n}\right)\in {\left[0,1\right]}^{m},\;\forall t_{n}\in \left[0,1\right]$. This also simplifies identifying suitable failure patterns by restricting the search space to a positive range.

With these assumptions in place, we propose an iterative approach that, in each iteration, does the following:

1. Generates a pseudo random failure pattern with a duration $K_{O}$;
2. Looks for occurrences of that failure pattern in the entire series of failure amplitudes, whatever the scale of the occurrence;
3. If some occurrences are found, then a new failure type is identified and the occurrences are associated with it;
4. The observations in each failure pattern occurrence are removed from the set of failure amplitudes, that is, the initial failure amplitudes are reduced according to the identified occurrences of the failure pattern;
5. The duration $K_{O}$ is decreased and a new iteration takes place;
6. If $K_{O}=1$, that is, if the pattern being searched corresponds to a single observation, then it will match all the remaining failure amplitudes, which will be grouped in a single failure type and interpreted as Noise.

While this brief enumeration of the iteration steps provides a global perspective of the approach, we now explain these steps in more detail.

In the first step, we generate a pseudo-random failure pattern $p_{n}\left(t_{n}\right)$ of a fixed duration $K_{O}$. The initial value of $K_{O}$ has to be provided by the user, who knows the application context and hence the possible maximum duration of a failure pattern. Failure patterns are generated from a set of more usually observed patterns, and they are reshaped using an evolutionary algorithm [40] while the series of failure amplitudes is being searched (in the second step) and similar patterns are found.

**Figure 8:**
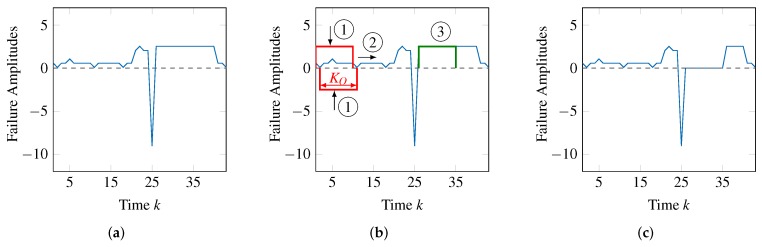
Steps for identifying failure types within the first phase of the processing chain. (**a**) initial failure amplitudes $f(k,o_{k})$; (**b**) step 2 for identifying failure types; (**c**) step 4 for identifying failure types.

To search for a pattern in the second step, the whole series of failure amplitudes is sequentially parsed, shifting the pattern matching window of $K_{O}$ observations (time steps) one observation at a time. This is illustrated in Figure 8b, where the pattern matching window with size $K_{O}$ (noted with ➀) is shifted to the right (noted with ➁). Therefore, at each step, $K_{O}$ observations are compared with the failure pattern, which is scaled (the value of $m_{n}\left(k,o_{k}\right)$ is determined) positively and negatively to match the corresponding failure amplitudes. This scaling, illustrated by vertical arrows, is also shown in Figure 8b at position ➀. Only if the pattern matches the failure amplitudes sufficiently, an occurrence of the failure type (time steps at which $s_{n}\left(k,o_{k}\right)=1$) is identified. Since the pattern is shifted through the time steps of $f(k,o_{k})$, if some occurrences exist, they will be found.

If some occurrences are found, then, in the third step of the iterative approach, the information relative to all these occurrences will be associated to a failure type so that it will be used in the second phase of the processing chain.

In the fourth step of the iterative approach, the idea is to remove the amplitudes corresponding to the occurrences of the failure pattern from the initial series of failure amplitudes. This way, in the next iteration, different failure types may be identified, contributing to the remaining failure amplitudes. However, it may be the case that the identified failure type is not significant. This happens if it includes a very small number of occurrences or if these occurrences have a very small scaling. In this case, the failure type is ignored and the respective occurrences are not removed from the initial series of failure amplitudes. Figure 8c also illustrates this step. The failure patterns noted with ➂ in Figure 8b are removed from the series of failure amplitudes, which becomes as shown in Figure 8c.

At the end of each iteration, the fixed duration $K_{O}$, with which the evolutionary algorithm searches for occurrences of a suitable failure type, is decreased. On the one hand, this limits the number of iterations the algorithm is required to finish. On the other hand, the last duration, with which the evolutionary algorithm is applied, is $K_{O}=1$. At this point, a failure pattern $p_{n}\left(t_{n}\right)=1$ is assumed. As a consequence, the pattern can be scaled to match each of the remaining failure amplitudes by setting $m_{n}\left(k,o_{k}\right)=f\left(k,o_{k}\right)$. Essentially, this means that the remaining failure amplitudes constitute occurrences of a failure type that may be interpreted as Noise.

### 5.2. Generalizing Failure Types

The output of the iterative algorithm of the previous phase is $F_{1}$, a set of $|F_{1}|=N_{1}$ failure types, each described by a failure pattern $p_{n}\left(t_{n}\right)$, its occurrences (time steps *k*) in $f(k,o_{k})$, and the scaling value $m_{n}\left(k,o_{k}\right)$ of each occurrence. Given the initial assumption that the lengths of duration of all occurrences of a single failure type are fixed to a constant value $K_{O}$, some information in $F_{1}$ might be redundant. This is the case when two failure types represented separately in $F_{1}$ have similar failure patterns and only differ in the duration of their occurrences. In this case, a single failure type could represent both by exploiting the fact that the *Time to Repair* may vary following a time- and value-correlated random distribution.

Therefore, in this section, we introduce the second phase of the processing chain, *Generalizing Failure Types*. It aims at identifying pairs of failure types ($\left(F_{y},F_{z}\right)$) that are representable by a single, combined failure type ($F_{\left\{y,z\right\}}$). By replacing the original failure types $F_{y}$ and $F_{z}$ with the combined failure type $F_{\left\{y,z\right\}}$, redundancy is reduced and the failure types are generalized. This generalization also means that the simplifying assumptions made in the first phase no longer have any significance or impact.

In brief, an iterative approach is proposed that consists of the following. In each iteration, every pair of failure types is combined, forming a new tentative failure type. Then, since the combination of two failure types leads to some intrinsic loss of information, this loss is measured for all pairs. Finally, if some combination is found implying an acceptably small loss, the original failure types are replaced by the new one.

To combine two failure types, the occurrences of the original failure types $F_{y}$ and $F_{z}$ are superimposed to determine the occurrences of the combined failure type $F_{\left\{y,z\right\}}$. As explained in Section 4.2, the occurrences of a failure type may vary regarding their scaling $m_{n}\left(k,o_{k}\right)$, but the failure pattern $p_{n}\left(t_{n}\right)$ must be the same. Therefore, given that the failure patterns of $F_{y}$ and $F_{z}$ may be different, or they may lead to different patterns when being superimposed, it is necessary to determine a new failure pattern that resembles, as much as possible, the superimposed pattern. For this, an RBF network is trained with all occurrences of the combined failure type $F_{\left\{y,z\right\}}$, thus representing this new pattern.

Given the input set $F_{1}$ of failure types, the generalization starts with assuming that the output set is the same as the input one, $F_{2}=F_{1}$. The described combination is thus applied to all the pairs in $F_{2}$. After that, it is then necessary to measure the information loss induced by each combination, for which we introduce $\epsilon_{\left\{y,z\right\}}$:

(11) $$\epsilon_{\left\{y,z\right\}}=\sum_{k=1}^{K}\left|f_{F_{\left\{y,z\right\}}}-f_{\left\{F_{y},F_{z}\right\}}\right|.$$

Here, $f_{\left\{F_{y},F_{z}\right\}}$ denotes a series of failure amplitudes, similar to the initial series, but containing solely the occurrences of the failure types $F_{y}$ and $F_{z}$. On the other hand, the series $f_{F_{\left\{y,z\right\}}}$ is constructed from the combined failure type, represented by the RBF network. For each time step in the series, a value for $f_{F_{\left\{y,z\right\}}}$ is calculated from the RBF network if the pattern is active in that time step. The resulting $\epsilon_{\left\{y,z\right\}}$ will be zero if the combined failure type faithfully represents the initial pair of failure types, that is, without loss of information. Otherwise, $\epsilon_{\left\{y,z\right\}}$ will be greater than zero.

Using $\epsilon_{\left\{y,z\right\}}$, we define a stopping criteria for generalizing failure types by restricting the loss of information to $\epsilon_{\left\{y,z\right\}}\leq E$. In that manner, E is nothing but a threshold for restricting the loss of information.

With this criteria, it is then possible to replace the original failure types by the combined failure type. For that, the minimal assessment value $min_{\epsilon_{\left\{y,z\right\}}}$ over all pairs of failure types is determined. In case the loss of information is acceptable, that is, $min_{\epsilon_{\left\{y,z\right\}}}\leq E$, the original failure types $F_{y}$ and $F_{z}$ are removed from $F_{2}$ while their combined failure type $F_{\left\{y,z\right\}}$ is appended to $F_{2}$.

In summary, by applying this iterative process, the number of failure types $|F_{2}|=N_{2}$ is reduced by one in each iteration. Furthermore, by accepting the combined failure type only if the caused loss of information is less than a threshold E ($min_{\epsilon_{\left\{y,z\right\}}}\leq E$), the maximal loss of information is limited. As a result of the generalization, the intermediate set $F_{1}$ is transformed into the second intermediate set $F_{2}$ with a reduced number of failure types ($N_{2}\leq N_{1}$).

It must also be noted that, after this generalization phase, the failure patterns $p_{n}\left(t_{n}\right)$ for each failure type in $F_{2}$ are already represented by an RBF network. Furthermore, the pattern $p_{n}\left(t_{n}\right)$ is now defined in the range $p_{n}\left(t_{n}\right)\in \left[-1,1\right],\;t_{n}\in \left[0,1\right]$ and, as the superimposed occurrences may vary in their duration, $d_{n}\left(k,o_{k}\right)$ of the combined failure type may vary too.

### 5.3. Parameterizing Failure Types

The previous phase produced the set $F_{2}$ containing all failure types comprising the final failure model. However, the failure types in this set are not yet fully represented by RBF networks (except for the failure patterns $p_{n}\left(t_{n}\right)$), but in terms of individual occurrences relative to each failure type. Therefore, this phase aims at completing the parameterization of the failure types regarding their activation ($a_{n}\left(k,o_{k}\right)$), deactivation ($d_{n}\left(k,o_{k}\right)$) and the scaling ($m_{n}\left(k,o_{k}\right)$).

All three of these functions are represented by time- and value-correlated random distributions (see Section 4.2.1) and each of these random distributions is modeled using an ICDF and time- and value-correlated mean and standard deviation functions (i.e., three functions). Therefore, a total of nine RBF networks must be trained to represent the activation, deactivation and the scaling functions.

To briefly explain which training data is necessary and how it is obtained, we consider the specific case of the RBF network representing the standard deviation $\sigma_{a}\left(k,o_{k}\right)$ of the activation function $a_{n}\left(k,o_{k}\right)$. In this case, the basic measure of interest is the Time Between Failures (TBF) for the failure type under consideration. While it is possible to obtain measures of the TBF from the failure type occurrences and calculate an overall standard deviation relative to those measures, simply doing that does not inform us about how the standard deviation is correlated with the time *k* and with the value $o_{k}$. What needs to be done is to partition the time and the value space into small ranges and obtain measures of interest (in this case the standard deviation of the TBF) within those ranges. Then, for training the RBF network, $\left(k,o_{k}\right)$ pairs (corresponding to the center of the considered ranges) will be used as input, while the corresponding value of interest (the standard deviation in that range) will be used as the output.

This reasoning has to be applied to all the time- and value-correlated functions to be represented by RBF networks. Concerning the representation of the random distribution using an ICDF, which is not time- or value-correlated, this can be done by considering the normalized values of interest in all ranges and determining the corresponding inverse cumulative distribution function.

**Figure 9:**
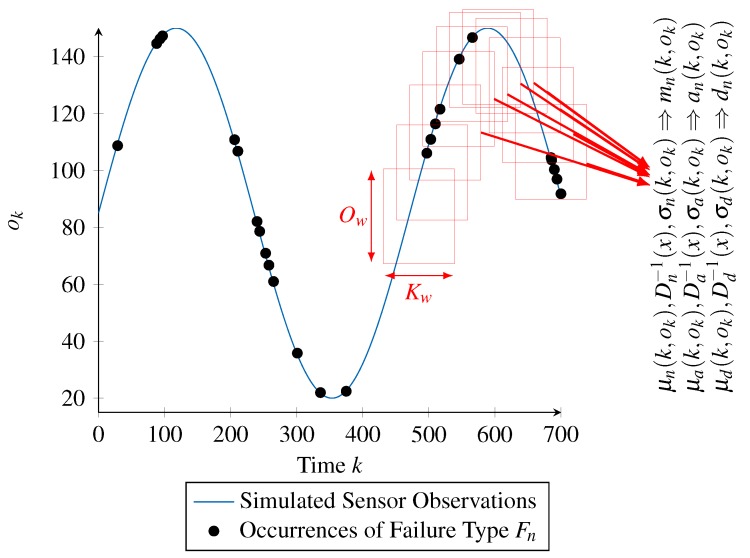
Sliding window approach for identifying time- and value-correlations for a failure type $F_{n}\in F_{2}$.

In a more generic way, the idea is to use a sliding window approach, with each window corresponding to the mentioned time and value range. This sliding window approach is illustrated in Figure 9 and is detailed ahead.

The approach starts by associating the occurrences of a single failure type (depicted by the black dots in Figure 9) to the series of reference values $o_{k}$ (blue curve) obtained as an input to the processing chain. From this representation, we start the sliding window approach. For that, a window is defined as a range of $K_{w}$ time steps as well as a range of $O_{w}$ sensor values, corresponding to the red rectangles in Figure 9. By shifting the window along the time axis with a step width of $K_{s}$ and along the value axis with a step width of $O_{s}$, multiple subsets of the failure type’s occurrences are generated.

The generated subsets form the base on which the training data for parameterizing the RBF networks is calculated. Training data for supervised learning, as it is the case with RBF networks, consists of pairs of input and target values. The input values required for the time- and value-correlated functions (mean and standard deviation of the scaling function $m_{n}\left(k,o_{k}\right)$, activation function $a_{n}\left(k,o_{k}\right)$ and deactivation function $d_{n}\left(k,o_{k}\right)$) are already given by the sliding window approach, since the subsets are associated with a time step *k* and a sensor value $o_{k}$ for the corresponding window.

However, the target values, that is, the intended outputs of the functions, need to be determined. These values are dictated by the definition of a time- and value-correlated random distribution. According to this definition, the values of the mean ($\mu_{Y}\left(k,o_{k}\right)$) and standard deviation ($\sigma_{Y}\left(k,o_{k}\right)$) are calculated using the Z-score normalization. Consequently, for the mean $\mu_{a}\left(k,o_{k}\right)$ and standard deviation $\sigma_{a}\left(k,o_{k}\right)$ of the activation function $a_{n}\left(k,o_{k}\right)$, we calculate the mean and standard deviation of the time between two occurrences of the failure type within each subset. Likewise, the target values of the functions $\mu_{d}\left(k,o_{k}\right)$ and $\sigma_{d}\left(k,o_{k}\right)$ are calculated by considering the duration of the occurrences within each subset, while the target values of $\mu_{n}\left(k,o_{k}\right)$ and $\sigma_{n}\left(k,o_{k}\right)$ are calculated by considering the scaling values of the occurrences within each subset (also illustrated in Figure 9).

Finally, as explained in Section 4.2.1, the remaining functions ($D_{a}^{-1}\left(x\right),\;D_{d}^{-1}\left(x\right),\;D_{n}^{-1}\left(x\right)$) representing the normalized random distributions are not correlated to the time *k* or value $o_{k}$. Therefore, training data for these functions is generated by considering the normalized values of all subsets and determining the corresponding inverse cumulative distribution function.

By extracting this data, we can train the RBF networks to represent the individual functions of a failure type $F_{n}\in F_{2}$. In that way, the parameter matrices of all RBF networks are determined and the parameter sets $PS_{A}$, $PS_{D}$ and $PS_{F}$ for each failure type are generated. As these form the final failure model, the output of the processing chain is constructed.

## 6. Evaluation Using an Infra-Red Distance Sensor

To evaluate the introduced approach for generic failure modeling of sensor failures, and also to evaluate the proposed processing chain for automatically generating a failure model, in this section, we consider a real infra-red distance sensor and we conduct an extensive experimental analysis to show the following: firstly, that the processing chain is able to extract appropriate failure models and hence can be used as a valuable tool for automating the process of obtaining failure characteristics of any one-dimensional sensor; secondly, that the generated failure models are able to capture particular failure characteristics in a better way than any other approach that we know of, fulfilling the requirements for being used in cooperative sensor-based systems.

Section 6.1 discusses the experimental setup and the generation of the failure model. For comparison, we additionally parameterize a normal distribution, uniform distribution, inverse cumulative distribution function, and a neural network to represent the sensor’s failure characteristics. To assess the performance of the generated failure models and to compare them with each other, Section 6.2 introduces two assessment measures. While both are based on the statistic of the Kolmogorov–Smirnov (KS) hypothesis test [42], each focuses on a different aspect. The first assesses the overall fit of a failure model with the sensor’s failure characteristics, while the second focuses on the representation of failure amplitudes with high magnitudes. In particular, the second aspect is of special interest when it comes to safety. We discuss the results of applying the assessment measures to the designed failure models in subsection 6.3.

### 6.1. Experimental Setup

To apply our methodology to real sensor data, an appropriate data acquisition is required. In this endeavor, we firstly describe the experimental setup using a Sharp GP2D12 [7,8] infra-red distance sensor, for acquiring sensor observations ${\hat{o}}_{k}$ relative to reference values $o_{k}$. Then, we provide details on using the obtained data for designing the envisioned failure models. Finally, to facilitate a subsequent comparison (in Section 6.2) between the designed failure models, we generate series of failure amplitudes by performing Monte Carlo simulations.

#### 6.1.1. Data Acquisition

**Figure 10:**
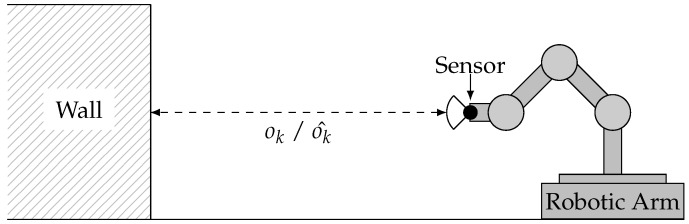
Evaluation setup to acquire real sensor data from a Sharp GP2D12 infra-red distance sensor.

For the envisioned evaluation, real sensor observations ${\hat{o}}_{k}$ of a Sharp GP2D12 infra-red distance sensor and corresponding reference values $o_{k}$ are obtained by mounting the distance sensor on a robotic arm and bringing it into defined distances to a wall, as illustrated in Figure 10.

The arm was moved into five different distances ($o_{k}\in \left\{56.5,51.5,43,31.5,21\right\}$ in cm), which are measured manually to obtain the ground truth. In each of them, 50,000 observations are acquired with a periodicity of 39 ms while not changing the sensor’s distance to the wall.

To furthermore facilitate designing and validating failure models, we use the first half of the observations for training data (on which we can apply the processing chain) and the second half for validation data. Therefore, a total of 125,000 observations for each, training and validation data, are obtained.

**Figure 11:**
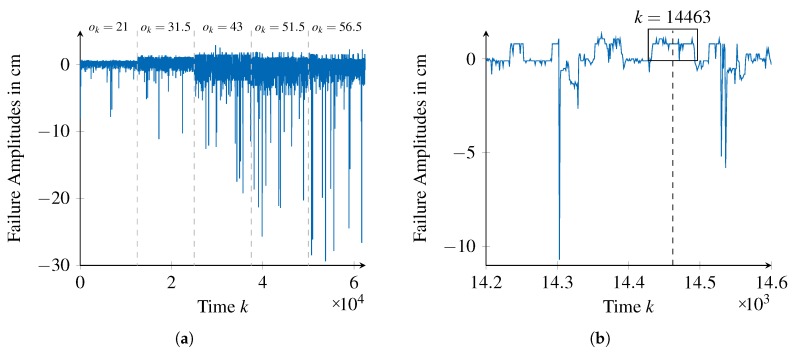
Failure amplitudes from the Sharp GP2D12 infra-red distance sensor. (**a**) complete series of failure amplitudes; (**b**) segment of the failure amplitudes for reference distance $o_{k}=31.5$ cm.

By calculating the difference between each observation and the reference value (ground truth), as stated in Equation (1) to the data, the failure amplitudes shown exemplarily in Figure 11 are obtained.

While Figure 11a provides an overview on the failure amplitudes of the sensor for all the considered reference distances (data for each distance is shown in temporal sequence but were acquired as independent runs), Figure 11b provides a segment of failure amplitudes for the distance of $o_{k}=43$ cm.

#### 6.1.2. Designing Failure Models

The obtained failure amplitudes and reference values enable us to design the envisioned failure models. We firstly apply the processing chain to extract a generic failure model before we discuss the parameterization of the traditional techniques (normal distribution, uniform distribution, ICDF). Finally, we provide details on the training of a feed-forward neural network [40]. By training it to represent a time- and value-correlated ICDF, we enable the comparison of the generic failure model with another approach capable of representing such correlations.

For extracting a generic failure model from the obtained training data, we configure the processing chain as follows:

**Identifying Failure Types:** This phase is applied twice to facilitate identifying failure types with different failure patterns. At first, we apply the phase on the failure amplitudes $f^{\prime }\left(k,o_{k}\right)$ smoothed by a median filter with a window size of $K_{w}=25$:
  (12) $$f^{\prime }\left(k,o_{k}\right)=median\left(\left\{f\left(k_{0},o_{k_{0}}\right)|k_{0}\in \left[k-\frac{K_{w}}{2},k+\frac{K_{w}}{2}\right]\right\}\right).$$
  In this way, constant failure patterns, exemplarily shown in Figure 11b around time step k= 14,463, can be identified. However, due to the smoothing constant, failure patterns in $f^{\prime }\left(k,o_{k}\right)$ that endure less than 50 time steps may deviate inappropriately from the actual failure amplitudes in $f(k,o_{k})$. Consequently, we fix the pattern length $K_{O}$ to be in the range of [50,180], which means that the search will start for patterns with length $K_{O}=180$, down to patterns with length $K_{O}=50$. This search results in 21 failure types being identified, explaining 12.4% of all the failure amplitudes.
  When applying this phase for the second time, we consider the unfiltered failure amplitudes $f(k,o_{k})$, from which we remove the occurrences of the failure types identified in the first application. Furthermore, we configure the pattern length $K_{O}$ to be in the range of [1,60]. The appropriateness of this choice was confirmed later, as only failure types with $K_{O}\leq 44$ could be found within the second search. From this search, 25 additional failure types with $K_{O}>1$ were identified, explaining 63.9% of the failure amplitudes. One last failure type, corresponding to $K_{O}=1$, accounts for the remaining failure amplitudes (23.7%). In summary, a total of 47 failure types were identified, which are passed to the next phase.
**Generalizing Failure Types:** The first parameter of this phase is the number of neurons of the RBF networks used to model the failure pattern $p_{n}\left(t_{n}\right)$. We set this parameter to 15 as the correspondingly trained networks yield acceptable error values while restricting the complexity of the training process. The second parameter is the stopping criteria E, which we set to 250 cm. This limits the loss of information caused by a single combination of failure types to 0.2% of the failure amplitudes of its corresponding original failure types. Therefore, the combined failure type represents the original failure types almost perfectly. With this parameterization, 29 of 47 failure types were combined, effectively reducing the number of failure types to 18. These were evaluated to explain 98.8% of the initial failure amplitudes, which means that the generalization caused an overall loss of information of 1.2%.
**Parameterizing Failure Types:** We configure the sliding window approach used within this phase with a window size of $K_{w}=2000$ and a step size of $K_{s}=100$. As we have 12,500 observations available for each reference value $o_{k}$, this configuration enables identifying potential time-correlations. Likewise, to facilitate the identification of value-correlations, we set the window size in the value domain to $O_{w}=4$ cm and the step size to $O_{s}=1$ cm. Given the measurement range of the distance sensor ($o_{k}\in$ [10 cm, 80 cm]), this configurations enables the identification of value-correlations in fine granularity.

Using this configuration, the processing chain extracts from the training data a parameterized generic failure model comprising 18 failure types.

For comparison, we parameterize a normal distribution N(μ,σ) to represent the same failure characteristics, as this is a frequently reported approach for failure characterization [12,15,16]. Using the training data, its mean μ and standard deviation σ is calculated over all time steps *k*.

A more restrictive approach is to solely state the minimal and maximal failure amplitudes [43,44]. Given that in this case there is no information about the distribution of failure amplitudes (within the minimal and maximal amplitudes), we assume a uniform distribution U(a,b) and calculate $a=min(f\left(k,o_{k}\right))$ and $b=max(f\left(k,o_{k}\right))$ using the failure amplitudes of the training data.

Similarly to both previous approaches, one can model the failure amplitudes using an inverse cumulative distribution function (ICDF) [45]. We obtain its parameterization by integrating the distribution function of the failure amplitudes in the training data and by inverting the result.

In contrast to our proposed approach, these approaches are established means for stochastic modeling, but are not capable of explicitly representing time- or value-correlations. Therefore, to provide a more fair comparison and evaluation of our approach, we train a traditional feed-forward neural network [40] to represent a time- and value-correlated inverse cumulative distribution function. In this endeavor, we calculate the inverse cumulative distribution function for each distance ($o_{k}\in \left\{56.5,51.5,43,31.5,21\right\}$ in cm) within the training data and associate it with the corresponding reference value $o_{k}$ and the time *k*. By sampling the obtained ICDFs to generate training data for the neural network, it learns not a static ICDF, but adjusts it corresponding to the provided time *k* and reference value $o_{k}$.

#### 6.1.3. Monte Carlo Simulation of Failure Models

The several failure models that we constructed in the previous section are not directly comparable with each other, that is, it is not possible to know how much better or worse they represent the failure characteristics of the distance sensor just by comparing the parameters describing them. Therefore, to support a comparison, in this section we sample the previously obtained failure models using Monte Carlo simulations. The obtained series of failure amplitudes, one for each failure model, constitute expressions of what the failure model actually represents. Therefore, they allow their comparison by observing how closely the respective failure amplitudes resemble the ones originally obtained from the distance sensor. They also facilitate a comparison with the validation data (i.e., the originally obtained failure amplitudes that were not used for obtaining the failure models), which is done in Section 6.3 using the assessment measures introduced in Section 6.2.

The generation of a series of failure amplitudes using a Monte Carlo simulation is directly supported by the generic failure model as a consequence of using inverse cumulative distribution functions within time- and value-correlated random distributions, as expressed in Equation (3). Three arguments are required to evaluate this equation: a uniformly distributed random value x∈U(0,1) to evaluate the ICDF ($D_{Y}^{-1}\left(x\right)$), a time step *k*, and a value $o_{k}$ to evaluate the mean $\mu_{Y}\left(k,o_{k}\right)$ and standard deviation $\sigma_{Y}\left(k,o_{k}\right)$. While *x* is generated by a random number generator, time *k* as well as the reference value $o_{k}$ can be taken from the series of validation data obtained in Section 6.1.1. The same inputs are required for the Monte Carlo simulation of the feed-forward neural network as it is trained to represent a time- and value-correlated ICDF.

On the other hand, since the approach representing the exact ICDF does not model the time- and value-correlations, a Monte Carlo simulation of this approach requires only uniformly distributed random numbers x∈U(0,1). In a similar way, Monte Carlo simulations for the normal distribution and the uniform distribution are facilitated by drawing random numbers from their respective standard distributions and scaling them afterwards. In the case of the normal distributions, a random number $x_{n}\in N\left(0,1\right)$ is scaled according to $f_{N}\left(k\right)=x_{n}\cdot \sigma +\mu$ while a random number x∈U(0,1) is scaled according to $f_{U}\left(k\right)=x\cdot \left(b-a\right)+a$ for sampling the uniform distributions.

**Figure 12:**
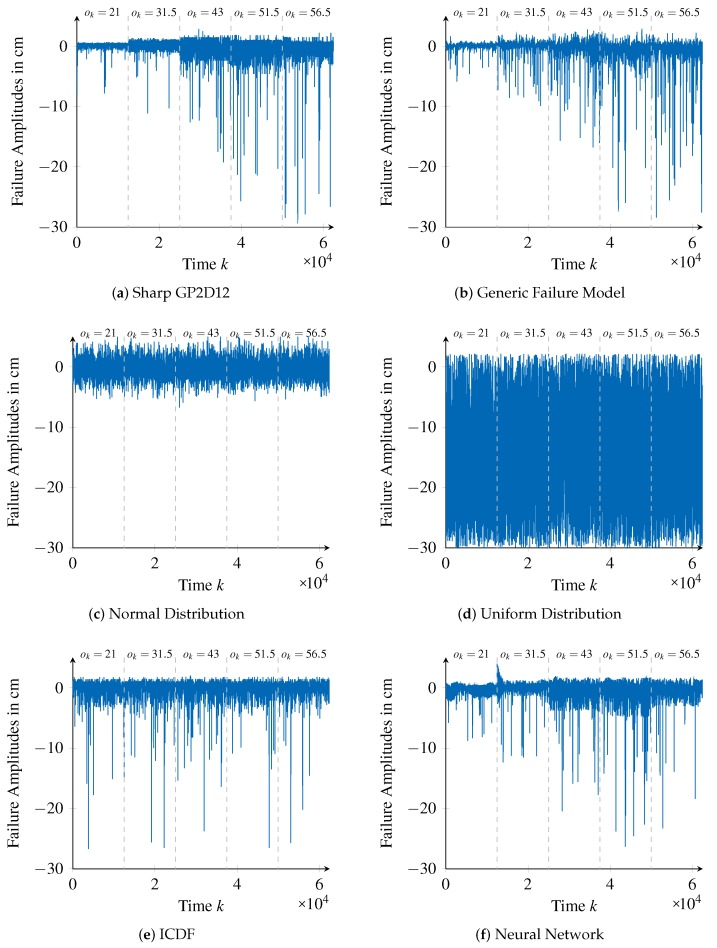
Failure amplitudes generated by different failure models to represent the failure characteristics of the Sharp GP2D12 infra-red distance sensor.

The generated failure amplitudes of different failure models are shown in Figure 12 along with the validation data (Figure 12a).

### 6.2. Introducing Assessment Measures Based on the Kolmogorov–Smirnov Statistic

For comparing the series of failure amplitudes generated in the previous section with the failure amplitudes of the distance sensor, we introduce two assessment measures. The first one considers the the overall match between the validation data and a failure model, in order to evaluate its appropriateness in general. The second is defined to specifically assess the appropriateness of the failure models with respect to safety concerns. Given that in this respect is is fundamental to ensure that worst case characteristics are well represented, the second measure focuses on the representation of failure amplitudes with high magnitudes.

#### 6.2.1. Assessing the Goodness of Fit on Average

Due to their nature, failure amplitudes are subject to randomness, which prohibits comparing two series of failure amplitudes directly, time step by time step. Instead, we apply a stochastic approach, the Kolmogorov–Smirnov (KS) statistic [42]. The statistic is used as a basis for the KS hypothesis test that is commonly applied to test whether or not two random variables *H* and *G* are following the same distribution. In that endeavor, the KS statistic compares their cumulative distribution functions $D_{H}\left(x\right)$ and $D_{G}\left(x\right)$ by calculating the maximal differences between them:

(13) $$d_{ks}=max_{x}\left|D_{H}\left(x\right)-D_{G}\left(x\right)\right|.$$

As this statistic does not restrict the underlaying distributions of *H* or *G* and can be applied to empirical data sets, it is well-suited for comparing the series of failure amplitudes in this evaluation. However, as it considers only the maximal difference between two cumulative distribution functions, only a global statement about the considered series of failure amplitudes is provided. In contrast, the failure amplitudes of the distance sensor exhibit time- and value-correlations, as visible in Figure 12a. To assess whether or not these are represented by the individual failure models, we need to adapt the measure to generate a more local statement.

We thus apply a sliding window approach similar to the one described in Section 5.3 so that, instead of comparing all failure amplitudes of *H* and *G* at once, we consider only failure amplitudes within a local interval covering $K_{w}$ time steps. By calculating the value of $d_{ks}$ over the failure amplitudes in this window, a local statement about the goodness of fit is obtained and time- or value-correlations are considered. To successively cover all failure amplitudes, the window is shifted through time by a step width of $K_{s}$. As this generates a value of $d_{ks}$ for each window, another series of assessment values is calculated. For the sake of simplicity, we sacrifice some of the locality of the statement by averaging over all calculated values of $d_{ks}$. In this way, a scalar value $\bar{d_{ks_{1}}}$ assessing the fit of a failure model with the validation data is calculated.

#### 6.2.2. Assessing the Goodness of Fit for Failure Amplitudes with High Magnitudes

The previously introduced measure assesses the goodness of fit considering all failure amplitudes within a certain time range. It therefore assesses to which degree a failure model matches the time- and value-correlations of failure characteristics in general. However, when it comes to safety, the representation of failure amplitudes with high magnitudes by a failure model is even more important. To explicitly assess this property, we adapt the first measure to consider only failure amplitudes with high magnitudes.

The idea is to filter the failure amplitudes for each window within the sliding window approach, for which we utilize the *Three Sigma Rule* [46]. This rule states that 99.74% of a normally distributed random variable’s values are within the range of [μ−3·σ,μ+3·σ]. Consequently, values outside this range can be considered to have a high magnitude. Although the failure amplitudes of the considered Sharp sensor are not normally distributed, this rule still provides appropriate criteria for deciding whether or not failure amplitudes have a high magnitude, at least for the purpose of the intended evaluation. Nevertheless, we relax this criteria by a factor of 2, meaning that a higher number of high failure amplitudes will be considered for the KS statistic and hence the assessment will be more encompassing with respect to the intended safety-related purpose. We also use the sliding window approach to produce a series of assessment values, which we average to obtain the final value of $\bar{d_{ks_{2}}}$.

With both measures in place, we can assess the goodness of fit of the designed failure models regarding the validation data. Furthermore, by comparing the assessment values between the different failure models, a comparison between them is facilitated.

### 6.3. Results

To calculate the proposed assessment values for the designed failure models, the underlying sliding window approach needs to be parameterized regarding its window size $K_{w}$ and step size $K_{s}$. As the validation data comprises 12,500 observations for each reference value $o_{k}$, a window size of $K_{w}=$ 12,500 is appropriate. However, to show that the effect of varying window sizes is limited and the conclusions drawn from the assessment values are therefore robust to it, we vary the values of $K_{w}\in \{1562,3125,6250,$ 12,500}. Furthermore, we set the step size to $K_{s}=500$, which ensures overlapping windows while maintaining significant changes between subsequent windows. The obtained assessment values $\bar{d_{ks_{1}}}$ and $\bar{d_{ks_{2}}}$ are listed in Table 2.

The considered failure models are listed row-wise while the varying window sizes $K_{w}$ are listed column-wise. Each cell holds either the assessment value $\bar{d_{ks_{1}}}$ (for the first four columns) or $\bar{d_{ks_{2}}}$ (for the last four columns). Values close to zero indicate well-fitting failure models while higher values imply the opposite. For reference, we also list the assessment values obtained by comparing the training data with the validation data in the first row (white cells). Furthermore, for a visual comparison, we colored the cells as follows. The cells associated with the generic failure model are colored blue. Cells holding assessment values better (lower) than the corresponding value of the generic failure model are colored green, while cells with worse (higher) assessment values are colored red. In the single case of equality, the cell is colored yellow.

To compare the proposed generic failure model approach with the remaining ones, the failure amplitudes presented in Figure 12 provide an important visual complement to the assessment values $\bar{d_{ks_{1}}}$ and $\bar{d_{ks_{2}}}$ listed in Table 2. Therefore, we often refer to this figure in the following discussion.

The approach using the uniform distribution is clearly the worst one. This is because only the minimal and maximal possible failure amplitudes are considered and hence the distribution of failure amplitudes in between is not represented. Therefore, the distribution of the validation data is not met, which is also shown in Figure 12d. Furthermore, in this case, no value-correlations are represented, which can also be seen in Figure 12d. Concretely, neither the value-correlated increase of the variance of frequently occurring failure amplitudes nor the value-correlated increase of high magnitudes of infrequently occurring failure amplitudes are represented by the uniform distribution approach.

When comparing the generic failure model and the normal distribution approaches, $\bar{d_{ks_{1}}}$ values show that they provide similar performances. However, this is only with respect to the representation of frequently occurring failure amplitudes. In this case, the normal distribution’s mean μ and standard deviation σ are calculated to match failure amplitudes on average, thus leading to good results. On the other hand, the bad performance of the normal distribution approach to represent rarely occurring failure amplitudes with high magnitudes is not only made evident by the assessment values of $\bar{d_{ks_{2}}}$, but also by Figure 12c.

In contrast to the normal distribution approach, the approach using an ICDF represents the exact distribution of failure amplitudes of the training data. Therefore, it achieves better assessment values compared to the generic failure model for the first assessment measure $\bar{d_{ks_{1}}}$. However, the results compare worse when considering the second assessment measure $\bar{d_{ks_{2}}}$. This means that this approach is not as good in representing high (and rare) failure amplitudes, which is particularly visible when considering the smallest window size ($K_{w}=1562$), for which inability to represent the value-correlation of higher failure amplitudes is exacerbated. This inability becomes apparent in Figure 12b, where it is possible to see that failure amplitudes with high magnitudes occur unrelated to the reference value $o_{k}$.

It is interesting to observe the results relative to the neural network approach, which, differently from the ICDF approach, are supposedly able to correctly represent time- and value-correlations. However, the values of $\bar{d_{ks_{2}}}$ for the neural network underline that failure amplitudes with high magnitudes are still not represented as well as by the generic failure model. Furthermore, increasing the window size $K_{w}$ plays favorably to our approach, whose performance is almost not affected. The performance degradation of the neural network approach for higher window sizes may be explained by uncertainties and artifacts introduced during the training of the network, also with relevance when considering high failure amplitudes. For instance, some deviations towards the positive range of failure amplitudes in the beginning of $o_{k}=31.5$, visible in Figure 12c, end up degrading the assessment values for windows including this range. If few large windows are considered for calculating the final assessment value (which is averaged over all windows), then a single artifact will have a more significant impact.

When considering $\bar{d_{ks_{1}}}$ values, the neural network approach performs sightly better than our approach. This indicates a better fit of frequently occurring failure amplitudes, which is underlined by Figure 12c. Still, from a safety perspective, we believe that out approach is better as it provides almost the same performance as the neural network with respect to $\bar{d_{ks_{1}}}$ assessment values, while it performs significantly better when it comes to represent failures with high amplitudes.

Nevertheless, in both cases, it is likely that optimizing the training procedure for designing a neural network, as well as optimizing the configuration and usage of the processing chain for extracting a generic failure model, enables better results.

**Figure 13:**
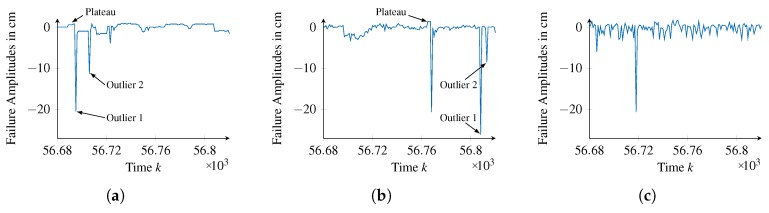
Comparing 140 time steps of failure amplitudes from the Sharp GP2D12 sensor, the generic failure model, and the neural network. (**a**) validation data; (**b**) generic failure model; (**c**) neural network.

A more fundamental difference between both approaches becomes apparent when comparing their generated series of failure amplitudes with the validation data at a detailed level, which are shown in Figure 13.

At about time step 56,690, the validation data show a small plateau before decreasing severely and thereby forming an Outlier. A similar shape can be found within the failure amplitudes of the generic failure model, near time step 56,770. Contrarily, the failure amplitudes of the neural network are exhibiting Outliers, but lack the plateau before. Furthermore, while the validation data and the generic failure model show two Outliers in sequence, as illustrated in Figure 13d,e, a similar pattern can not be found in the data of the neural network. Although these are very particular examples, they clarify that the generic failure model is capable of representing the time behavior of failure amplitudes using failure pattern $p_{n}\left(t_{n}\right)$, whereas the neural network solely represents their distribution.

Finally, we can compare both approaches with respect to the requirements defined in Section 2. As the *Generality*, *Clarity*, and *Coverage* requirements are fulfilled by both approaches due to their use of neural networks and mathematical expressions, the *Comparability* requirement is of most interest. With respect to the neural network, this requirement is only partially fulfilled. For comparing a failure model, the neural network has to be sampled to either obtain an application specific representation of the ICDF or a Monte Carlo simulation has to be performed. To obtain any further information, an application has to apply corresponding calculations. On the other hand, the generic failure model supports more flexible evaluations. For instance, for calculating the maximal duration of a failure type, only the deactivation function has to be evaluated. Similarly, to determine the average scaling of a failure type, only the mean function $\mu (k,o_{k})$ has to be evaluated. Finally, when performing a Monte Carlo simulation on the generic failure model, not only a series of failure amplitudes is obtained, but also detailed information about the activation, deactivation and failure amplitudes of individual failure types is acquired. This flexibility renders the *Comparability* requirement to be fulfilled by the generic failure model.

## 7. Conclusions

The work presented in this paper is motivated by the question on how to maintain safety in *dynamically composed systems*. As an answer, we proposed an integration step to take place in the application context, for analyzing at run-time the failure model of an external sensor with respect to the application’s fault tolerance capacities. By rejecting or integrating the external sensor’s observations depending on the outcome of this run-time safety analysis, the safety of *dynamically composed systems* is maintained.

For applying this concept, we identified four requirements (*Generality*, *Coverage*, *Clarity* and *Comparability*) that have to be fulfilled by appropriate failure models of external sensors. In addition, by reviewing the state of the art on sensor failure modeling in different research areas, we showed that no current approach meets the listed requirements.

Then, as a fundamental novel contribution of this paper, we introduced a mathematically defined, generic failure model. It utilizes not only the concept of failure types, but also explicitly supports representing time- and value-correlated random distributions. As the second major contribution, we introduced a processing chain capable of automatically extracting appropriate failure models from a series of failure amplitudes.

To validate both contributions, we used the processing chain to extract a generic failure model for representing the failure characteristics of a Sharp GP2D12 infra-red distance sensor, which we then used to perform a detailed comparative analysis with a set of other approaches. This comparative analysis underlined the applicability of the approach as well as the fulfillment of the predefined requirements.

Nevertheless, the generic failure model was evaluated solely with respect to one-dimensional failure characteristics. To confirm the fulfillment of the *Coverage* requirement and simultaneously facilitate the adoption of the generic failure model, representing failure characteristics of multidimensional sensors is required in future work. Similarly, while *Comparability* is given due to the structure and composition of the proposed failure model, an empirical evaluation with respect to a real dynamically composed system is planned. In this manner, the representation of an application’s fault tolerance using the same methodology shall be investigated and an approach for matching it with a failure model shall be determined. This will underline not only the fulfillment of this requirement, but also the applicability of the proposed integration step.

Furthermore, to increase the applicability of the generic failure model to safety-critical systems in general, the processing chain shall be extended in such a way that certain properties (e.g., completeness, no under-estimation of failure characteristics) for extracted failure models can be guaranteed.

Besides dynamically composed systems, versatile applications of the generic failure model are feasible. Due to its mathematical and structural definition, its interpretation is not only clear, but can be automated too. Therefore, using it for automatically parameterizing appropriate failure detectors and filters facilitates a promising research direction too. This idea can be extended to the usage of the generic failure model within approaches for online monitoring of the quality of sensor observations, e.g., the validity concept [47].

## Figures and Tables

**Table 1:** Overview on the fulfillment of the predefined requirements by individual approaches of the state of the art on sensor failure modeling.

Research Area	Reference	Generality	Coverage	Clarity	Comparability
Fault Injection	Saraoğlu et al. [12]	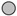	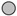	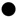	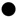
	Reiter et al. [13]	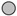	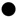	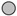	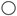
Sensor Networks	Ni et al. [14]	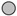	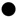	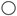	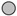
	Sharma et al. [15]	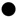	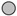	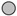	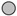
	Elnahraway et al. [16]	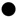	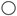	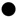	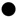
	Sheng et al. [17]	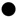	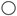	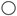	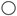
	Urteaga et al. [18]	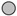	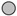	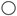	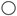
Fault Detection and Isolation (FDI)	Dai et al. [19]	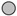	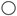	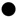	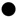
	Balaban et al. [20]	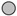	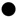	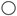	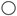
	Hereida et al. [21]	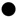	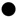	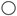	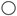
	Zug et al. [4]	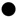	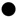	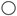	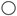
Depth Cameras	Foix et al. [22]	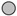	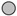	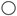	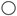
	Koshelham et al. [23]	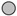	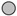	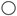	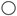
	Höbel et al. [24]	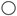	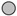	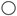	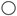

Legend: 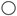
—not fulfilled, 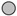
—partially fulfilled, 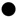
—fulfilled.

**Table 2:** Values of $\bar{d_{ks_{1}}}$ and $\bar{d_{ks_{2}}}$ for failure amplitudes of the Sharp sensor with varying window sizes $K_{w}$.

	$K_{w}$	1562	3125	6250	12,500	1562	3125	6250	12,500
**Failure Model**									
Training Data		0.086	0.070	0.057	0.045	0.463	0.488	0.492	0.496
Generic		0.291	0.283	0.274	0.258	0.498	0.498	0.500	0.500
U(a,b)		0.840	0.839	0.836	0.832	0.605	0.600	0.594	0.582
N(μ,σ)		0.286	0.281	0.277	0.268	0.738	0.733	0.732	0.739
ICDF		0.201	0.194	0.183	0.162	0.589	0.575	0.567	0.533
Neural Network		0.291	0.274	0.254	0.232	0.520	0.531	0.548	0.606
		$\bar{d_{ks_{1}}}$				$\bar{d_{ks_{2}}}$			

## References

[1] Birolini A. (2017). Reliability Engineering.

[2] Ruijters E., Stoelinga M. (2015). Fault tree analysis: A survey of the state-of-the-art in modeling, analysis and tools. Comput. Sci. Rev..

[3] Isermann R. (2006). Fault-Diagnosis Systems: An Introduction from Fault Detection to Fault Tolerance.

[4] Zug S., Dietrich A., Kaiser J. (2012). Fault Diagnosis in Robotic and Industrial Systems.

[5] Lee J., Bagheri B., Kao H.A. (2015). A cyber-physical systems architecture for industry 4.0-based manufacturing systems. Manuf. Lett..

[6] Whitmore A., Agarwal A., Da Xu L. (2015). The Internet of Things—A survey of topics and trends. Inf. Syst. Front..

[7] SHARP Cooperation GP2D12 Data Sheet, 2005. http://www.sharpsma.com/webfm_send/1203.

[8] SHARP Cooperation GP2D12 Optoelectronic Device. https://engineering.purdue.edu/ME588/SpecSheets/sharp_gp2d12.pdf.

[9] Kabadayi S., Pridgen A., Julien C. (2006). Virtual Sensors: Abstracting Data from Physical Sensors. Proceedings of the 2006 International Symposium on on World of Wireless, Mobile and Multimedia Networks.

[10] Frank R. (2000). Understanding Smart Sensors.

[11] Willke T.L., Tientrakool P., Maxemchuk N.F. (2009). A survey of inter-vehicle communication protocols and their applications. IEEE Commun. Surv. Tutor..

[12] Saraoğlu M., Morozov A., Söylemez M.T., Janschek K., Tonetta S., Schoitsch E., Bitsch F. (2017). ErrorSim: A Tool for Error Propagation Analysis of Simulink Models. Computer Safety, Reliability, and Security: 36th International Conference, SAFECOMP 2017, Trento, Italy, 13–15 September 2017, Proceedings.

[13] Reiter S., Viehl A., Bringmann O., Rosenstiel W. Fault injection ecosystem for assisted safety validation of automotive systems. Proceedings of the 2016 IEEE International High Level Design Validation and Test Workshop (HLDVT).

[14] Ni K., Ramanathan N., Chehade M.N.H., Balzano L., Nair S., Zahedi S., Kohler E., Pottie G., Hansen M., Srivastava M. (2009). Sensor Network Data Fault Types. ACM Trans. Sen. Netw..

[15] Sharma A.B., Golubchik L., Govindan R. (2010). Sensor Faults: Detection Methods and Prevalence in Real-world Datasets. ACM Trans. Sen. Netw..

[16] Elnahrawy E., Nath B. (2003). Cleaning and Querying Noisy Sensors. Proceedings of the 2nd ACM International Conference on Wireless Sensor Networks and Applications.

[17] Sheng B., Li Q., Mao W., Jin W. (2007). Outlier Detection in Sensor Networks. Proceedings of the 8th ACM International Symposium on Mobile Ad Hoc Networking and Computing.

[18] Urteaga I., Barnhart K., Han Q. REDFLAG a Run-timE, Distributed, Flexible, Lightweight, In addition, Generic fault detection service for data-driven wireless sensor applications. Proceedings of the 2009 IEEE International Conference on Pervasive Computing and Communications.

[19] Dai X., Qin F., Gao Z., Pan K., Busawon K. Model-based online sensor fault detection in Wireless Sensor Actuator Networks. Proceedings of the 2015 IEEE 13th International Conference on Industrial Informatics (INDIN).

[20] Balaban E., Saxena A., Bansal P., Goebel K.F., Curran S. (2009). Modeling, Detection, and Disambiguation of Sensor Faults for Aerospace Applications. IEEE Sens. J..

[21] Heredia G., Ollero A., Bejar M., Mahtani R. (2008). Sensor and actuator fault detection in small autonomous helicopters. Mechatronics.

[22] Foix S., Alenya G., Torras C. (2011). Lock-in Time-of-Flight (ToF) Cameras: A Survey. IEEE Sens. J..

[23] Khoshelham K., Elberink S.O. (2012). Accuracy and Resolution of Kinect Depth Data for Indoor Mapping Applications. Sensors.

[24] Höbel J., Jäger G., Zug S., Wendemuth A., Tonetta S., Schoitsch E., Bitsch F. (2017). Towards a Sensor Failure-Dependent Performance Adaptation Using the Validity Concept. Computer Safety, Reliability, and Security: 36th International Conference, SAFECOMP 2017, Trento, Italy, 13–15 September 2017, Proceedings.

[25] MATLAB/Simulink product description. https://www.mathworks.com/products/matlab.html.

[26] Bell R., International Electrotechnical Commission IEC 61508: Functional Safety of Electrical/Electronic/ Programmable Electronic Safety-Related Systems. Proceedings of the IEE Colloquium on Control of Major Accidents and Hazards Directive (COMAH)—Implications for Electrical and Control Engineers.

[27] System C Standardization Working Group 1666-2001-IEEE Standard for Standard SystemC Language Reference Manual. http://ieeexplore.ieee.org/servlet/opac?punumber=6134617.

[28] Reiter S., Viehl A., Bringmann O., Rosenstiel W. White-Box Error Effect Simulation for Assisted Safety Analysis. Proceedings of the 2015 Euromicro Conference on Digital System Design.

[29] Akyildiz I.F., Su W., Sankarasubramaniam Y., Cayirci E. (2002). A survey on sensor networks. IEEE Commun. Mag..

[30] Pinto A.M., Costa P., Moreira A.P., Rocha L.F., Veiga G., Moreira E. Evaluation of Depth Sensors for Robotic Applications. Proceedings of the 2015 IEEE International Conference on Autonomous Robot Systems and Competitions.

[31] Brade T., Zug S., Kaiser J. Validity-based failure algebra for distributed sensor systems. Proceedings of the 2013 IEEE 32nd International Symposium on Reliable Distributed Systems (SRDS).

[32] Hussain S., Mokhtar M., Howe J.M. (2015). Sensor Failure Detection, Identification, and Accommodation Using Fully Connected Cascade Neural Network. IEEE Trans. Ind. Electron..

[33] Campa G., Fravolini M.L., Seanor B., Napolitano M.R., Gobbo D.D., Yu G., Gururajan S. (2002). On-line learning neural networks for sensor validation for the flight control system of a B777 research scale model. Int. J. Robust Nonlinear Control.

[34] Sharma A., Golubchik L., Govindan R. On the prevalence of sensor faults in real-world deployments. Proceedings of the 4th Annual IEEE Communications Society Conference on Sensor, Mesh and Ad Hoc Communications and Networks.

[35] Fortuna L., Graziani S., Rizzo A., Xibilia M.G. (2007). Soft Sensors for Monitoring and Control of Industrial Processes.

[36] Gilchrist W. (2000). Statistical modelling with quantile functions.

[37] Al Shalabi L., Shaaban Z., Kasasbeh B. (2006). Data mining: A preprocessing engine. J. Comput. Sci..

[38] Friedman J., Hastie T., Tibshirani R. (2008). The Elements of Statistical Learning.

[39] Ben-Daya M., Ait-Kadi D., Duffuaa S.O., Knezevic J., Raouf A. (2009). Handbook of Maintenance Management and Engineering.

[40] Kruse R., Borgelt C., Braune C., Mostaghim S., Steinbrecher M. (2016). Computational Intelligence: A Methodological Introduction.

[41] Park J., Sandberg I.W. (1991). Universal approximation using radial-basis-function networks. Neural Comput..

[42] Massey F.J. (1951). The Kolmogorov-Smirnov Test for Goodness of Fit. J. Am. Stat. Assoc..

[43] Song E., Lee K. (2008). Understanding IEEE 1451-Networked smart transducer interface standard-What is a smart transducer?. IEEE Instrum. Meas. Mag..

[44] Botts M., Robin A. (2007). OpenGIS^®^ Sensor Model Language (SensorML) Implementation Specification. OpenGIS Implement. Specif. OGC.

[45] Gentle J.E., Härdle W.K., Mori Y. (2012). Handbook of Computational Statistics: Concepts and Methods.

[46] Pukelsheim F. (1994). The Three Sigma Rule. Am. Stat..

[47] Brade T., Jäger G., Zug S., Kaiser J. (2014). Sensor-and Environment Dependent Performance Adaptation for Maintaining Safety Requirements. Computer Safety, Reliability, and Security.

